# Patient Navigation and Time to Diagnostic Resolution: Results for a Cluster Randomized Trial Evaluating the Efficacy of Patient Navigation among Patients with Breast Cancer Screening Abnormalities, Tampa, FL

**DOI:** 10.1371/journal.pone.0074542

**Published:** 2013-09-16

**Authors:** Ji-Hyun Lee, William Fulp, Kristen J. Wells, Cathy D. Meade, Ercilia Calcano, Richard Roetzheim

**Affiliations:** 1 H. Lee Moffitt Cancer Center & Research Institute, Tampa, Florida, United States of America; 2 University of South Florida, College of Medicine, Tampa, Florida, United States of America; 3 San Diego State University and Moore’s Cancer Center, San Diego, California, United States of America; Sudbury Regional Hospital, Canada

## Abstract

**Objectives:**

The objective of this study was to evaluate a patient navigation (PN) program that attempts to reduce the time between a breast cancer screening abnormality and definitive diagnosis among medically underserved populations of Tampa Bay, Florida.

**Methods:**

The Moffitt Patient Navigation Research Program conducted a cluster randomized design with 10 primary care clinics. Patients were navigated from time of a breast screening abnormality to diagnostic resolution. This paper examined the length of time between breast abnormality and definitive diagnosis, using a shared frailty Cox proportional hazard model to assess PN program effect.

**Results:**

1,039 patients were eligible for the study because of an abnormal breast cancer screening/clinical abnormality (494 navigated; 545 control). Analysis of PN effect by two time periods of resolution (0-3 months and > 3 months) showed a lagged effect of PN. For patients resolving in the first three months, the adjusted Hazard Ratio (aHR) was 0.85 (95% Confidence Interval [CI]: 0.64-1.13) suggesting that PN had no effect on resolution time during this period. Beyond three months, however, navigated patients resolved more quickly to diagnostic resolution compared with the control group (aHR 2.8, 95%CI: 1.30-6.13). The predicted aHR at 3 months was 1.2, which was not statistically significant, while PN had a significant positive effect beyond 4.7 months.

**Conclusions:**

PN programs may increase the timeliness of diagnostic resolution for patients with a breast cancer-related abnormality. PN did not speed diagnostic resolution during the initial three months of follow up but started to reduce time to diagnostic resolution after three months and showed a significant effect after 4.7 months.

**Trial Registration:**

ClinicalTrials.gov NCT00375024

## Introduction

Medically and historically underserved populations often experience delays in breast cancer diagnosis and treatment, more late-stage breast cancer diagnosis, and overall higher breast cancer-related mortality and morbidity [1-3]. Studies have found that diagnostic delays of three or more months can reduce survival in patients with breast cancer [4,5]. While detection and treatment of breast cancer in its early stage improves long term survival [6], timely diagnostic care following a symptom or screening abnormality can be impeded by several factors including personal, logistical, and health system barriers, as well as a lack of social support to obtain needed care [7-9].

Patient navigation (PN) is a patient-centered health care service delivery model that centers on reducing barriers to cancer care [10-13]. Most studies that have evaluated whether PN is associated with better adherence to recommended diagnostic care or more timely receipt of diagnostic care following an abnormal screening mammogram or symptom of breast cancer have found that PN is indeed a promising strategy [14-17]. However, some of these studies had limitations in research design, necessitating the conduct of larger, controlled trials of PN. The National Cancer Institute (NCI) and the American Cancer Society funded nine Patient Navigation Research Program (PNRP) sites across the United States to evaluate whether PN is associated with timely adherence to recommended cancer care [18-20]. The Moffitt Cancer Center PNRP (Moffitt PNRP) is one of the nine sites. The Moffitt PNRP was a cluster randomized trial to evaluate the efficacy of PN in improving timeliness of diagnostic resolution of cancer related abnormalities among a vulnerable, medically underserved population of racial and ethnic minorities and migrant farm workers in Tampa Bay, Florida.

The efforts and goals of the Moffitt PNRP were further enhanced through collaborative interactions with established community partners of the Tampa Bay Community Cancer Network (TBCCN). TBCCN represents a highly complementary NCI funded community network program, comprised of 22 community partners, designed to address the cancer burden in racial/ethnic minorities and other underserved across populations by engaging community members through Community-Based Participatory Research [21].

We reported that PN did not have a significant effect on median time to diagnostic resolution among a combined sample of patients with either breast or colorectal abnormality, using a general linear mixed model approach [19]. Because clinical care of persons with breast and colon abnormalities is quite different, it is important to understand if the effectiveness of PN differs in these two cancer conditions.

The objective of this study was to evaluate the efficacy of PN among patients enrolled in the Moffitt PNRP study as a result of a breast related abnormality using a more detailed analysis of time-to-diagnostic resolution. Because other studies have used less specific measures of the time to diagnostic resolution (i.e., diagnostic resolution within three months versus six months of abnormality), this investigation added new information by examining whether a more precise measurement of the timing of diagnostic resolution helped clarify the efficacy of the PN program.

## Methods

The protocol for this trial and supporting CONSORT checklist are available as supporting information; see Checklist S1 and Protocol S1. The Moffitt PNRP used a controlled cluster randomized trial (CRT) design in which clinics were randomized to either PN group or control group, and the outcomes and variables were measured on patients within the clinics. More detailed information about CRT (equivalently group randomized trial) design appears in Lee et al. (2009) [22].

### Clinic Recruitment and Randomization

Project investigators approached Tampa Bay health care organizations with community- based primary care clinics, serving populations affected by health disparities in both urban and rural areas. A total of 12 clinics from five health care organizations agreed to participate in the study. Randomization was stratified by health care organizations, as clinics within each health care organization were relatively homogeneous. Seven and five clinics were randomly assigned PN and control groups, respectively, and they joined the study between 2/27/2006 and 6/27/2008. Randomization was conducted by the study statistician using the procedure of PLAN in SAS.

### Participant Population and Sample

Similar to the demographic characteristics of the Tampa Bay region [23], the populations served by the primary care clinics in this study were mostly Hispanic, African-American, and White. Patients with an abnormality on clinical breast examination, mammography (BIRADS 0, 3, 4, 5), ultrasound, or magnetic resonance imaging that required additional diagnostic imaging or referral to a specialist for further evaluation were eligible to participate in the study. Participants were also considered eligible for PN if they had pathologically confirmed newly diagnosed breast cancer but had not yet undergone initial treatment. Cancer patients were not included in our analysis due to the primary outcome of time to definitive resolution. Patients were excluded if they were cognitively impaired, institutionalized, less than 18 years old, diagnosed with a previous cancer within the past five years (excluding non-melanoma skin cancer), or currently undergoing cancer treatment.

### Participant Identification and Recruitment

Participants were enrolled between 3/11/2006 and 12/15/2009. The last chart reviews were performed in 8/2010. Eligible patients were identified through several methods including mammography screening logs, information from referral coordinators, identification/referral from clinical staff, and computer searches of relevant diagnostic codes. The medical records of participants at control clinics were reviewed when it was determined that a patient met inclusion criteria. Once participants were identified at navigation clinics and a written referral was provided by the patient’s health care providers, a patient navigator contacted the patient and obtained informed consent for the study during an in-person visit. The PN program did not attempt to change referral patterns within participating clinics, and each clinic continued their usual referral pattern that included community hospitals, public hospitals, and a tertiary cancer center. Clinics generally referred patients to centers that were geographically close. Most clinics had a member of the office staff specifically assigned to help implement patient referrals. The study was approved by the Institutional Review Board of the University of South Florida. As participation of control patients was limited to medical record abstraction, informed consent was waived by the IRB.

### Patient Navigation (PN) Intervention

The nation’s first PN program was initiated by Harold Freeman at Harlem Hospital Center in New York City in 1990 in an effort to close the health disparity gap and to enhance the health experiences of patients [18,24]. Since then, the PN programs are being increasingly adopted across the nation. The goal of PN, as an intervention, is to promote the timely care of an individual patient in a culturally sensitive manner by eliminating barriers across all phases of the health care continuum. The barriers that PN program are attempting to overcome are specific to each patient navigated. Some common barriers include difficulty with communication, inadequate health literacy, and difficulties with arranging transportation or scheduling an appointment. Therefore, the core function of PN is achieved through a one-on-one relationship between the patient navigator and the patient. As one of nine national PNRP sites, the Moffitt PNRP was designed to evaluate the efficacy of PN in timeliness of diagnostic resolution for patients with breast or colorectal abnormalities. The PN program was evaluated in four counties among an ethnically and medically underserved population with breast or colorectal abnormalities in the Tampa Bay area of Florida. Five paid lay patient navigators provided PN services in the Moffitt PNRP [19].

### Control

Patients of clinics randomized to the control group did not receive services of a PN, but was provided usual medical care, which may include referral to specialty services for follow up of the breast cancer screening abnormality.

### Primary Study Outcome

The main outcome for this study (denoted as T1) was length of time between the abnormal symptom or screening date and date of definitive diagnosis. For patients who did not achieve definitive diagnosis, the date of last follow-up was recorded. Clinical follow-up of identified abnormalities occurred through 2/2010. Definitive diagnosis was defined as the point in time in which a breast cancer was diagnosed or a non-breast cancer diagnosis was rendered and no further immediate evaluation was required. The definitive diagnosis could result from biopsy, additional imaging, or other diagnostic tests, or by clinical assessment of a medical specialist.

### Recruited and Analytic Sample Sizes

**Figure 1:**
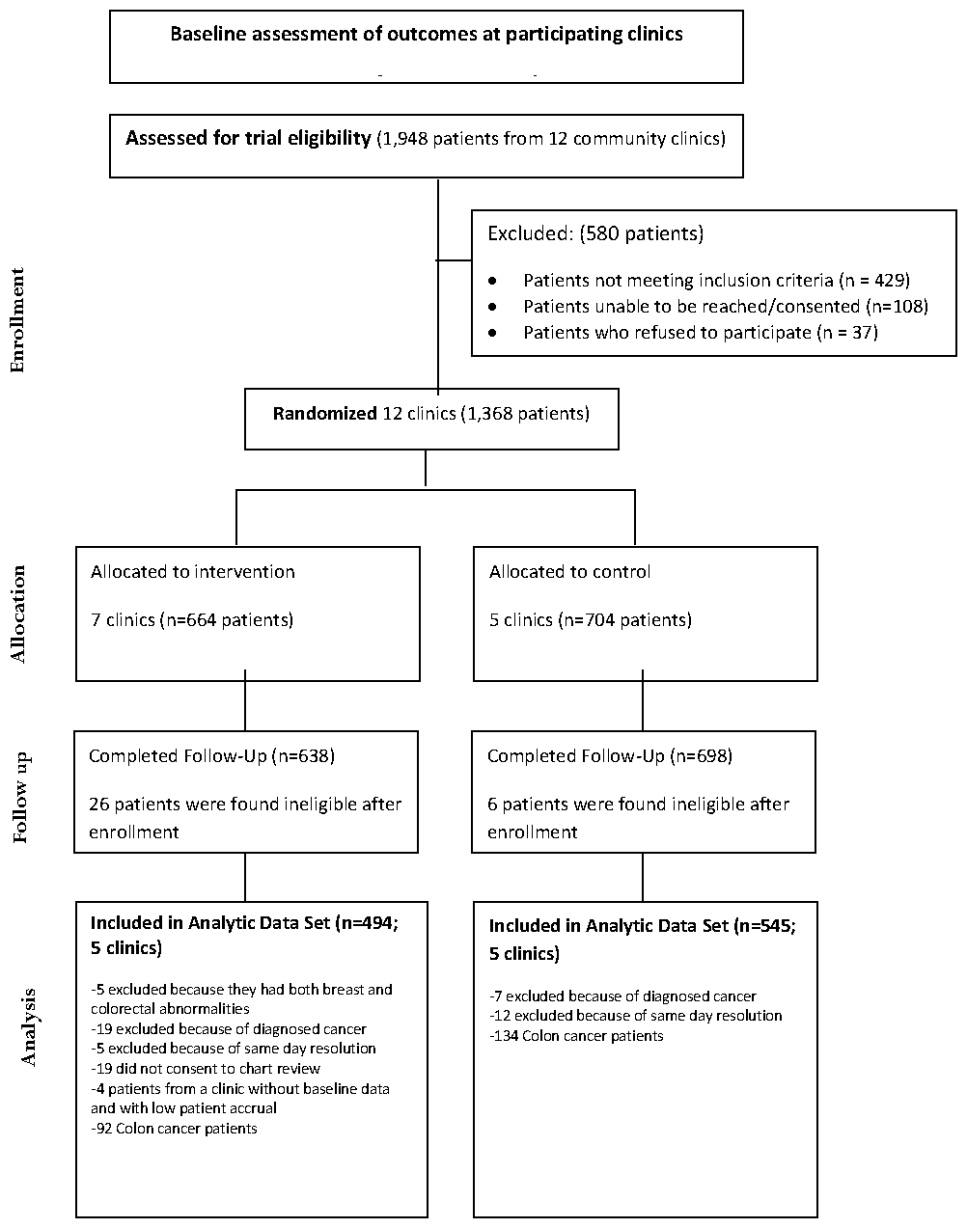
CONSORT flow diagram of Tampa PNRP and Patients with Breast. Cancer Screening Abnormalities. This is partially modified from the previous our study report for the different analytic data set (page 1665; Figure 1) [19].

Based on a priori sample size calculation, our protocol targeted 1,400 eligible patients at 12 clinics with an average of 111 participants from each clinic [25]. After assessing patients for trial eligibility, there were 1,368 patients randomized either to PN intervention or to usual care [19]. See CONSORT diagram (Figure 1) for enrollment, allocation, follow-up, and the final analytic participants having breast cancer abnormalities. For this study we excluded those patients who had colon cancer abnormality (n=226). After reviewing their charts after randomization, 32 patients were deemed ineligible and excluded. We additionally excluded the following patients: patients who had both breast and colorectal abnormalities (n=5), had diagnosed cancer (n=26), had resolved on the very day of their initial abnormality (n=17), did not provide a consent form to have their chart reviewed (n=19), or were from one of the two health care organizations having very few intervention patients to be navigated (n=4). The final analytic sample size included 494 participants from 5 clinics randomized to receive patient navigation and 545 participants from 5 control clinics.

### Statistical Analysis

Our analytic approach was based on the cluster randomized trial (CRT) design [22] of the study in which participants may be clustered by clinic. Demographic and clinical variables at baseline were compared between the groups, using the generalized linear mixed effects models in which the clinic was treated as a random effect.

Survival analysis approaches were used for the time-to-resolution outcome (T1) to assess the PN program effect. Participants who had not achieved definitive diagnostic resolution were censored at the time of last medical record abstraction. The median resolution time and resolution rate at a certain time point was summarized, using the Kaplan Meier method. An examination of the overall survival curves showed that the two curves crossed at 3 months. This implied the PN effect did not appear immediately after the inception of PN program. In addition, from a statistical point of view this implies that the constant proportional hazards assumption would be invalid for this data. Therefore, a stratified analysis was considered to examine the effect of patient navigation by two time periods: 0 -<= 3 months and > 3 months from screening abnormality to diagnostic resolution period.

Shared frailty Cox proportional hazards models were used to estimate the PN effect. Each clinic was a cluster that contributes multiple patients to the input data set. To account for the expected intraclass correlation among patients within a clinic, the clinic was treated as a normally distributed random effect using a shared frailty. We included the following covariates as potential confounders in the multivariable models; ethnicity (Hispanic, non-Hispanic), language (English, non-English), marital status (married, not-married), and insurance (some form of health insurance, uninsured). The proportional hazard assumption was tested using graphical and numerical methods [26]. The predicted hazard ratio of PN at a certain time point was calculated from a multiple Cox model using a linear combination of the main effect of PN and the interaction term between PN and time. The significance of each predicted hazard ratio across time was tested using a Wald test in the model. All tests were two-sided. Analyses were conducted using SAS (version 9.3; SAS Institute, Cary, NC).

## Results

### Participant Characteristics

Overall, most participants were female (99.0%), Hispanic (60.4%), non-English speakers (54.8%), had no health insurance coverage (54.2%), had less than a high school education (54.6%), and had household incomes of less than $20,000 per year (88.3%). About half the participants were married, with navigated patients being more likely to be married (58.7%) than control patients (42%) and having no more than an 8^th^ grade education (46.2%) compared to control patients (22.2%). Fewer navigated patients had a family history of breast cancer compared to control patients (4.5% vs. 8.8%) (Table 1). Participants were generally recommended to have either additional imaging (ultrasound 51.3%, diagnostic mammography 27.8%) or breast biopsy (17.4%) to determine whether or not they had breast cancer. For patients receiving PN, the median time from diagnostic abnormality to first contact with PN was 19 days.

**Table 1:** Demographic and Social Economic Characteristic of Patients by Groups at Baseline.

**Variables: Levels**	**Control Group N=545**	**Patient Navigation Group N=494**	**P value ^(1)^**
Age at diagnosis *in years*: Mean (STD)	47.6 (13.0)	41.0 (11.4)	0.28
Gender			
Female	537 (98.7%)	491 (99.4%)	0.41
Male	7 (1.3%)	3 (0.6%)	
Race			
Black non Hispanic	78 (14.9%)	24 (4.9%)	0.28
White non Hispanic	185 (35.4%)	84 (17.1%)	
Hispanic/Latina	234 (44.7%)	378 (77.0%)	
Mixed/Other non Hispanic	26 (5.0%)	5 (1.0%)	
Ethnicity			
Not Hispanic/Latina	289 (55.3%)	113 (23%)	0.18
Hispanic/Latina	234 (44.7%)	378 (77%)	
Language			
English	323 (60%)	142 (29%)	0.14
Non-English	215 (40%)	348 (71%)	
Marital Status			
Married	198 (41.8%)	256 (58.7%)	0.02
Non-Married	276 (58.2%)	180 (41.3%)	
Education			
8th grade or less	49 (22.2%)	144 (46.2%)	0.04
Some high school	38 (17.2%)	60 (19.2%)	
High school diploma(including equivalency)	68 (30.8%)	72 (23.1%)	
Some college/vocational after high school or Associate degree or College graduate	66 (29.9%)	36 (11.5%)	
Income			
Less than $10,000	191 (60.1%)	153 (40.9%)	0.12
$10,000 to $19,999	101 (31.8%)	166 (44.4%)	
$20,000 to $29,999	17 (5.3%)	47 (12.6%)	
$30,000 or more	9 (2.8%)	8 (2.1%)	
Employment			
Employed full time	94 (24.2%)	120 (30%)	0.65
Not employed full time	295 (75.8%)	280 (70%)	
Health Insurance Status			
Coverage	335 (62%)	135 (27.8%)	0.20
No health insurance coverage	205 (38%)	350 (72.2%)	
Insurance Type among Those Insured			
Private insurance	37 (11.1%)	17 (12.8%)	0.88
Medicaid(no private or Medicare)	85 (25.6%)	35 (26.3%)	
Medicare(no private)	56 (16.9%)	13 (9.8%)	
Other government insurance(no private, Medicare, or Medicaid)	154 (46.4%)	68 (51.1%)	
Family history of breast			
No	497 (91.2%)	472 (95.5%)	0.03
Yes	48 (8.8%)	22 (4.5%)	
Charlson Comorbidity Index score			
0	441 (80.9%)	436 (88.3%)	0.67
1	80 (14.7%)	52 (10.5%)	
2+	24 (4.4%)	6 (1.2%)	

(1) Generalized Mixed Effects Model P Value for Variable vs. Control/Navigated

### Characteristics of the Time to Diagnostic Resolution of the Breast Abnormality (T1)

**Figure 2:**
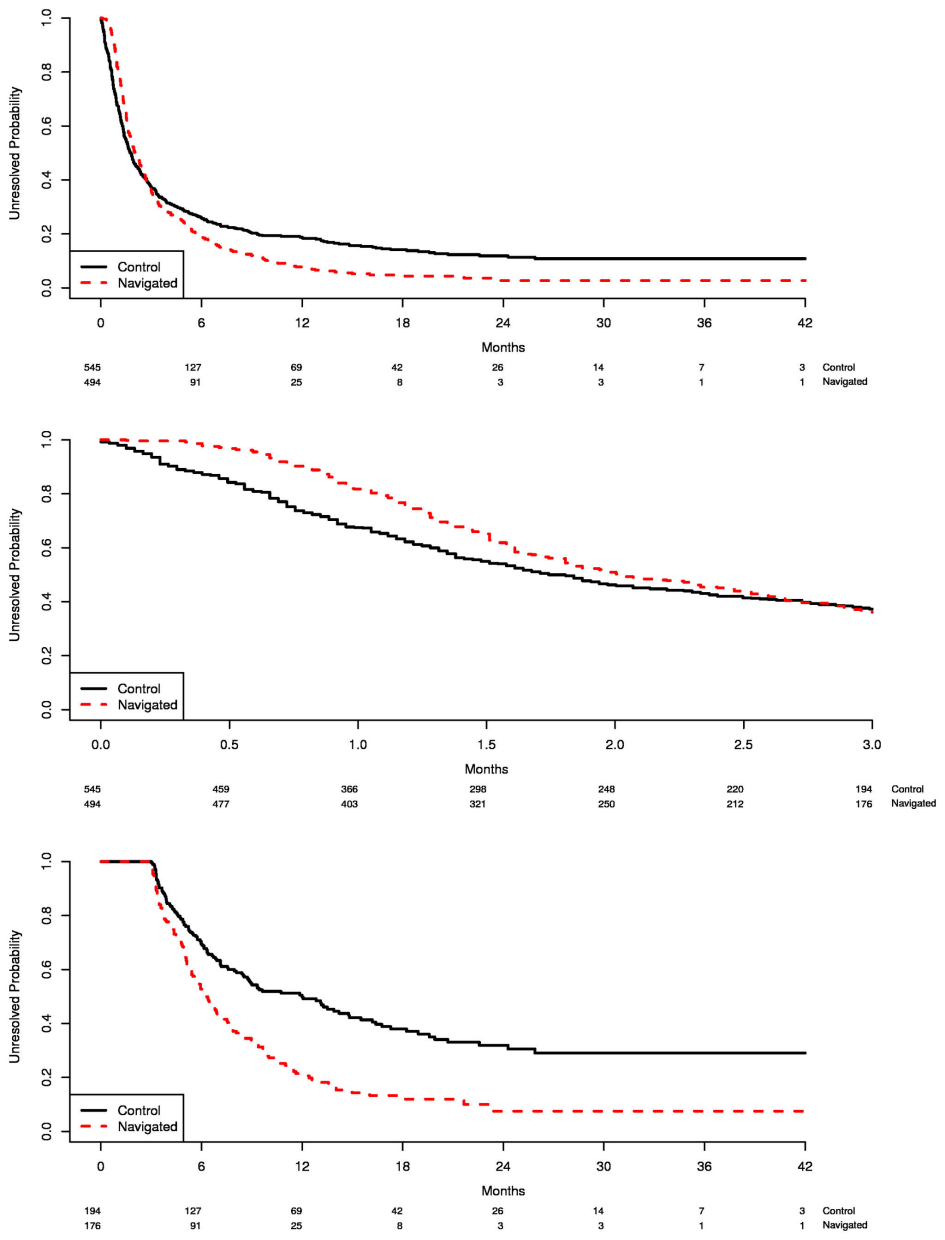
Kaplan-Meier estimates of time to diagnostic resolution: overall and 3 months cut-points. A: All patients across overall time period. B: Patients who resolved prior to 3 months. C: Patients who resolved beyond 3 months.

912 of the participants (88%) reached diagnostic resolution, and 127 (12%) were censored (i.e. never resolved). The distributions of time to diagnostic resolution (T1) were examined for all participants and the histogram showed an extremely skewed distribution. Kaplan-Meier curves suggested that the median time between the screening abnormality and diagnostic resolution was not different between the two groups, although the PN group had a longer median time than the control group (2.0 vs. 1.7 months). However, at about three months the survival curves of the two groups crossed, and the resolution rates showed a dramatic change. Specifically, prior to three months the control group appeared to have quicker diagnostic resolution than the PN group, but beyond three months, those receiving PN seemed more likely to achieve diagnostic resolution in less time (Figure 2a). This was also confirmed by checking the adequacy of the Cox regression model over time, i.e., the proportional hazard assumption for the Cox model was violated for the PN effect (p<0.0001).

#### PN effect during 0-3 months

All study participants (n=1039) were included in this time period analysis. Patients who resolved or were lost to follow-up after three months (n=15) were censored at 3 months. The proportion of patients who did not resolve at 1 month was 82% for PN group and 68% for control group (Figure 2b). The adjusted hazard ratio (aHR) was 0.85 (0.64-1.13), indicating the resolution rate for PN group was slower than the control group, although it was not statistically significant (Table 2 upper panel). Marital status was the only statistically significant variable in the model (married women had faster resolution rates, aHR=1.39 95% CI: 1.16-1.67).

**Table 2:** Multivariable analysis for time to diagnostic resolution among patients having breast cancer abnormal symptoms by the resolution time period (prior- and post-3 month).

Period	Variables	Adjusted Hazard Ratio^+^	95% CI
0 − <= 3 months	PN vs. Control	0.85	0.64-1.13
	Hispanic vs. Non-Hispanic	1.15	0.88-1.51
	English vs. Non-English	1.01	0.77-1.34
	Health Insurance vs. No Health Insurance	1.01	0.83-1.23
	Married vs. not-married	1.39	1.16-1.67
> 3 months			
	PN vs. Control	2.83	1.30-6.13
	Hispanic vs. Non- Hispanic	1.31	0.84-2.04
	English vs. Non-English	0.65	0.41-1.03
	Health Insurance vs. No Health Insurance	0.95	0.68-1.31
	Married vs. not-married	1.04	0.78-1.38

+ Four covariates were adjusted for in the model: Ethnicity, Language, Marital status, and Health Insurance.

#### PN effect between > 3 months and the last follow-up

For this time period analysis, patients who resolved on or before 3 months were excluded (n=669), leaving 370 participants in the analysis. Beyond three months, the PN group showed a significantly shorter median time to resolution compared to the control group: 6.2 months (95% CI: 5.5-7.1) and 12 months (95% CI: 8.5-14.8), respectively (Figure 2c). The resolution rate at 12 months was 79% and 50% for the PN and control groups, respectively. The aHR was 2.83 (95% CI: 1.30–6.13), indicating a significantly quicker resolution rate of PN compared to the controls (Table 2 lower panel).

#### PN effect on time-to-resolution from multivariable model across time

Table 3 shows the results of the multivariable shared frailty Cox analysis for T1 with an interaction term of PN with time as well as the other covariates used above. The main effect of PN was estimated with an aHR of 0.85 (95% CI: 0.57-1.29), which is the effect of PN when month was 0 or when patients first had the abnormal symptoms. The PN effect decreased the hazard of resolution by about 15%, although this was not statistically significant. The interaction term, PN x Time, was statistically significant at the 0.001 level, indicating that the effect of PN varies with time.

**Table 3:** Multivariable analysis for time to diagnostic resolution among patients having breast cancer abnormal symptoms for all participants.

Variables	Parameter Estimate	Standard Error	Adjusted Hazard Ratio (95% CI)^+^	p-value^+*^
Patient Navigation (PN)	-0.158	0.210	0.85 (0.57-1.29)	0.454
PN x Time	0.118	0.020	1.13 (1.08-1.17)	<0.001

+ Four covariates were adjusted for in the model: Ethnicity, Language, Marital status, and Health Insurance.

* The p-values were adjusted for the shared frailty random effect.

#### Predicted PN effect to time-to-resolution

**Figure 3:**
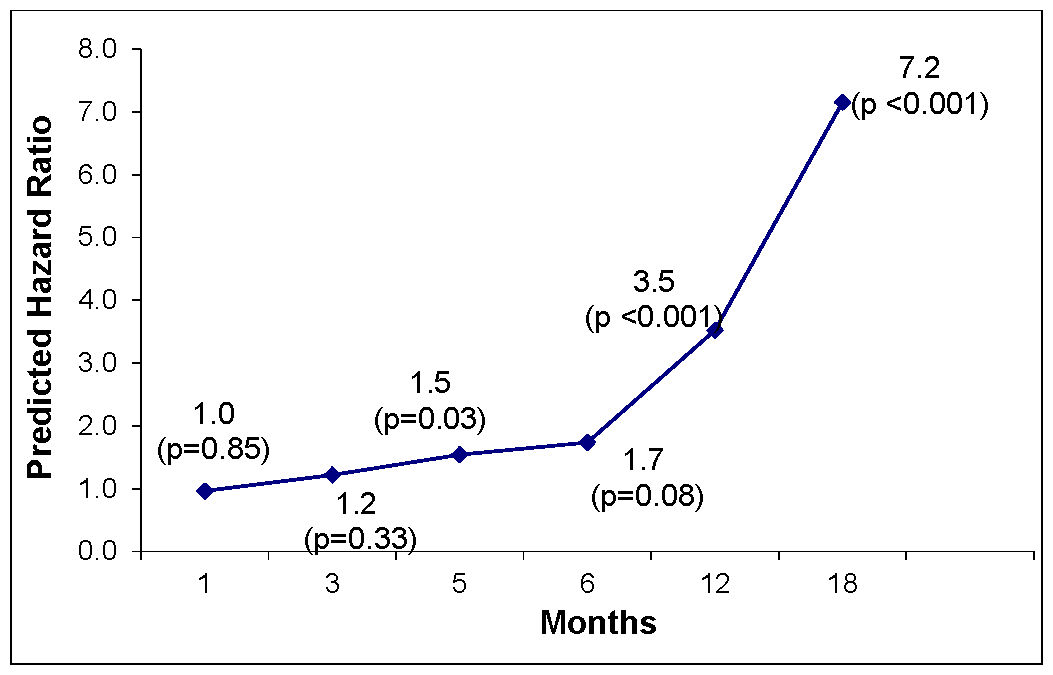
Predicted Hazard Ratio for PN across Time (Months) and P-value.

To investigate how the effect of PN changes over time we quantified the PN’s effect for any given month from the estimated Cox model as [-0.158 + 0.118 x Months] from Table 3. For example, at one month from the abnormal symptom, the PN effect is approximately 0. At three months the final PN effect, while controlling for other covariates, was 0.20 (aHR=1.2; p=0.33). At five months, the PN effect was 0.43 (aHR=1.5; p=0.034). Finally at 6 months and 12 months the aHR were calculated as 1.7 (p=0.008) and 3.5 (p=0.0001), respectively. Figure 3 illustrates the predicted aHRs across time. It appears that PN had no statistically significant effect on the hazard of resolution during the first 4.6 months but a significant positive effect beyond 4.7 months (p < 0.05).

## Discussion

Previous studies found inconsistent results about PN on the time to diagnostic resolution [6,14-17,27] ; while one study found that PN had no effect on the time to diagnostic resolution of a breast cancer screening abnormality [28], several other studies found that PN led to more timely diagnostic resolution [14-17,27]. The results from our study may explain these discrepancies. We found that PN exerted its significant and positive effect after a certain time point. Among participants who resolved beyond three months, those who received PN were more likely to achieve diagnostic resolution in less time than participants who received usual care. The benefit of PN, therefore, seems to begin occurring around three months, and by about 5 months, PN was superior to usual care in reducing the time to diagnostic resolution of the screening abnormality.

There are several possible reasons for the lack of navigation impact early in the follow up period. First, because this was a research study there were requirements to receive formal referral from primary care providers and obtain patient informed consent before navigation could begin. For one-third of navigated patients, formal navigation did not begin until more than one month after the initial abnormality. Navigators thus faced several logistic hurdles (awaiting formal referral, contacting and consenting patients) that were not present in the control group.

We found that the impact of PN was greater for persons with delayed diagnostic resolution and PN impact appeared to increase exponentially over follow-up time. Persons with delayed resolution may have substantial barriers to care and require greater assistance to reach resolution. Therefore, these patients having personal, logistical, and health system barriers may be the most likely to be lost to follow-up and in greatest need of patient navigation. Our data suggest that the greater the delays in diagnostic resolution the greater the impact of patient navigation. Future research is needed to better define which patients are most likely to benefit from PN so that this resource can be targeted.

Although not statistically significant, there were substantive socio-demographic differences (ethnicity, language, health insurance) between navigated and control patients that, based on prior research, would tend to favor the control arm [29-33]. We also found evidence that providers tended to selectively refer persons for PN that providers judged would have more difficulty reaching diagnostic resolution [19]. This may have biased our findings towards a null effect.

This study was conducted in clinics that are committed to improving the lives of medically underserved persons. The results of navigation may differ among other populations and in other settings. Because of our limited sample size, we did not examine other outcomes that are potentially important such as cancer stage at diagnosis, patient satisfaction, the effect of PN on treatment outcomes, and cost effectiveness. These outcomes will be reported separately by the national PNRP research group. Finally, our study focused on patients with breast cancer related abnormalities and the effects of PN may differ for other cancers.

In conclusion, the Moffitt PNRP found that PN reduced overall time from screening abnormality to diagnostic resolution for persons with delayed diagnosis. Our results also suggest that benefits of navigation increase as diagnostic delay increases. Further research is warranted to investigate the specific reasons that cause diagnostic delays.

## Supporting Information

Checklist S1 **CONSORT Checklist.**
(DOC)

Protocol S1 **Trial protocol.**
(DOC)
