# Supplementary material for: Patient Navigation and Time to Diagnostic Resolution: Results for a Cluster Randomized Trial Evaluating the Efficacy of Patient Navigation among Patients with Breast Cancer Screening Abnormalities, Tampa, FL
Source: PLoS One. 2013 Sep 16;8(9):e74542. doi: 10.1371/journal.pone.0074542 (PMC3774725; doi:10.1371/journal.pone.0074542)
Supplement: Protocol S1 — Trial protocol. (DOC) [file pone.0074542.s002.doc]

# Protocol Title: Patient Navigator Research Project

# Principal Investigator: Richard Roetzheim, MD

# Protocol Version Date: 12-18-09

# A. SPECIFIC AIMS

There have been unprecedented advances in translating research findings into public health practices to reduce cancer risks over the past few decades. Yet, such advances have not been realized by all members of society according to age, race, ethnic and socioeconomic groups [1-4]. Although survival rates have increased, significant challenges remain to close the gaps in cancer outcomes [5, 6]. The elimination of cancer disparities is a national imperative that requires the creation of accessible and beneficial interventions. The Tampa Bay region of west central Florida epitomizes this challenge, with low levels of education (20% without a high school education), high levels of poverty (12.5% living below the poverty line), a culturally diverse population (18% Hispanic and 15% African American), and high cancer incidence rates. This unfortunate combination translates to unacceptably high cancer mortality. Navigating through the medical care system can be difficult, thereby decreasing the likelihood of favorable outcomes and decreasing patient satisfaction.

Our **hypothesis** is that a culturally appropriate Patient Navigator Program can be implemented in the Tampa Bay region of Florida that will decrease delay in diagnosis and delivery of cancer care in an acceptable and cost-effective manner. The expectation is that this program will eventually lead to lower cancer mortality among underserved populations.

Our **approach** is predicated upon the assumption that strategies to promote early diagnosis and timely therapy of cancer are likely to improve survival. Our plan is based upon a partnership that includes an NCI designated comprehensive cancer center (H. Lee Moffitt Cancer Center & Research Institute), community hospitals and clinics, the American Cancer Society, and HealthChoice Network. Designed in partnership with community members, the investigative team represents expertise in community medicine, education, psychology, economics, and biostatistics. Through a group-randomized design, our **goal** is to evaluate the effectiveness of a Patient Navigator Program on several critical metrics, and disseminate to the broader scientific, medical, and public health community. The cancers we propose to specifically target are those of the breast and colorectum.

**Our aims are:**

1. Phase I: To work closely with our community partners to conduct formative research for the development of an acceptable, appealing, and appropriate Patient Navigator Program.

Approach: Through a multi-step and iterative process, we will finalize the initial plans developed in preparation for this application with our Community Advisory Board. This oversight group includes representatives of local hospitals, community clinics, the affiliate offices of the American Cancer Society, and a faith-based community health promotion organization (HealthChoice Network) that serve diverse communities. During Phase I we will also obtain baseline data, through chart abstraction, on the timeliness and cost of cancer care.

1. Phase II: To conduct a group randomized controlled trial to evaluate the Patient Navigator Program.

Approach: We will focus on patients diagnosed with a breast or colorectal cancer screening abnormality within our participating network. Four centers will receive the Navigator Program, and three will receive the intervention, but delayed. Data will be collected at the time of an abnormal screening finding, and at 1 month, 6 month, and 1 year to monitor timeliness of care, appropriateness of care, cost-effectiveness of the intervention, patient satisfaction and quality of life.

1. Phase III: To disseminate the findings from this research outside the Tampa Bay region.

Approach: We will use study outcome data, including results of cost-effectiveness analyses plus input from key stakeholders (participants and providers) to develop a plan for long-term dissemination and sustainability of the program.

This study is expected to result in a comprehensive evaluation of a Patient Navigator Program on quality of cancer care, patient satisfaction, quality of life and cost-effectiveness. Such knowledge is essential to improvements in reducing the mortality and suffering from cancer by 2015.

# B. BACKGROUND AND SIGNIFICANCE

Although the factors contributing to health disparities have been explored for other diseases such as cardiovascular disease, stroke and diabetes, the conditions that give rise to cancer health disparities have been less well studied [7]. As a result, measurable progress toward the 2010 goal has been less than favorable. We ascribe to the view that health and illness are inextricably tied to dynamics of social context, including people’s work and economic consequences, belief systems and educational achievements. As Freeman has stated, “Disease always occurs within the context of human circumstances, including social position, economic status, culture, and environment. To understand disparities in cancer incidence and outcome, there is a need to understand the circumstances in which they occur”[8]. The primary contribution to health disparities is the combination of discrimination, lack of resources and violation of human rights [2, 9]. Freeman notes that it is critical to identify which groups of Americans suffer a heavy burden of cancer, determine the cause and apply interventions to eliminate the disparities. Cancer morbidity and mortality disparities are the result of a complex interplay of low socioeconomic class, culture, social injustice and poverty[1, 9, 10]. For example, for all cancer sites combined, populations of low-income counties in the U.S. have higher cancer death rates than those found in more affluent counties. In addition, some ethnic groups (e.g., African Americans) have lower five-year survival rates than non-Hispanic whites [4]. In the following sections we will present a compelling rationale for the proposed study, including its focus on health disparities, the value of community participation in research, and why we have elected to focus on breast and colon cancers. Finally, we close with a summary of the importance of the expected results.

### **B.1 Magnitude of the Cancer Health Disparities Problem in Florida**

Although Florida residents experience similar illness burden and mortality as the general U.S. population, two important features distinguish the state: the demographic composition and the state economy. Together, these features give rise to a unique set of considerations for public health researchers and clinicians who want to reduce health disparities. Florida’s large population is diverse with regard to age, ethnicity and international immigration. These characteristics combine to shape the population’s general literacy level, English language proficiency and employment opportunities, which in turn are key variables that impact health status and access to health care.

Concerning the demographic profile, the state’s population was estimated to be more than 16.7 million in 2002, the fourth largest in the country [11]. As the gateway to Latin America and the Caribbean, Florida also is home to a growing and diverse foreign-born population (12.9 % in 1990, and 16.7% of the population in 2000 compared to 11.1% nationally)[11], with 72.8% of this population born in Latin America. Additionally, the state has a large proportion (17%) of persons aged 65 years or more [Kaiser, 2004]. With regard to the economy, Florida has sizeable tourism and agriculture industries. These provide many service-sector job opportunities, including low-wage and unskilled employment, that are often filled by low-literacy and low English proficiency workers. Such jobs frequently do not offer health benefits, thus contributing another barrier to accessing health care for low-income populations.

*B.1.1 The Racial/Ethnic Composition of the Tampa Bay Area* - The target population for the proposed Navigator Program includes Hillsborough county, which is the location of the Moffitt Cancer Center, the only NCI-designated Comprehensive Cancer Center based in Florida. Hillsborough County contains both urban and rural areas with an estimated population of 1,053,864 (2002 estimate)[11]. While the majority of the population (roughly 75%) is white, the county also is home to approximately 180,000 Hispanics, 150,000 Black/African Americans and 13,000 Caribbeans. It should be noted that census figures traditionally underestimate certain segments of the population, such as seasonal farm workers, because of factors such as immigration status and housing. Further, we acknowledge that the census categories reflect social constructions, not biological realities. We also recognize that persons from Latin America and the Caribbean who are of African descent may be categorized as “Black/African American.”

Additionally, the populations of our county area are ethnically and linguistically diverse and include a growing foreign-born population. Ethnic minority populations that are foreign-born have lower rates for cancer screenings than whites or U.S.-born ethnic minorities. Lower screening use may be attributable to factors such as insurance status (underinsured or uninsured) and linguistic or knowledge barriers about preventive health behaviors [12-14]. Latin American and Caribbean persons comprise the majority of immigrants; in Hillsborough County 11.5% of the population is foreign-born. Moreover, 35.6% of Hispanics and 60.6% of Caribbeans are foreign-born [11]. *Thus, the target population for the proposed study has the necessary degree of racial and ethnic diversity to examine the impact of a Patient Navigation Program.*

*B.1.2. Socioeconomic status of the Tampa Bay region* - Although educational attainment categories in the census do not allow us to separate out those with very low educational levels (e.g., those with less than nine years of schooling), we can note that the educational attainment for the population 25 years of age and older varies by ethnicity and the proportion of those with less than a ninth-grade education is considerably higher among Hispanics and African Americans in the tri-county area. According to the U.S. Census (2000) in Hillsborough County 18.2% of Hispanics have less than a 9^th^ grade education, compared to 3.3% of whites and 7.5% of African Americans. Poor as well as uninsured individuals are more likely to have cancer diagnosed at a later stage and are more likely to die of cancer [4]. A significant fraction of Hillsborough county lives below the poverty line: 26% of Blacks or African Americans, 19% of Hispanics, 21% of migrants from the Caribbean, and19% of those who report two or more races as their heritage. These poverty rates are higher than whites in the county and the national average. According to the Agency for Health Care Administration, there is a larger proportion of Hispanics and African Americans under the age of 65 who have no health insurance compared with whites in the area[15]. *The high prevalence of poverty and low education in the Tampa Bay region make it at especially high risk for health disparities.*

*B.1.3 Cancer morbidity and mortality in the Tampa Bay region* - Although all-cause morbidity and mortality rates in Florida are similar to the general U.S. population, cancer morbidity and mortality rates differ. For example, the breast cancer mortality rate in Florida is the third highest in the nation [16]. Even though the average annual age-adjusted death rates for breast and colorectal cancer are lower for Hispanics compared to other ethnic groups, the rates are higher for Hispanics in Florida than the national Hispanic average cancer death rates[17]. In 1999, cancer accounted for 24% of all deaths in Florida [18]. According to the Florida Cancer Data System (FCDS), the top three cancers diagnosed in women in the Tampa Bay area (defined by FCDS as Hillsborough, Pasco, Pinellas and Manatee County) area were breast, lung/bronchus, and colorectal. The top three cancers diagnosed in men in the area were prostate, lung/bronchus, and colorectal [19]. There is considerable racial disparity in these rates. For example, in Hillsborough County the cancer death rate for all sites combined for African American men was 47% higher than for white men. For all cancer sites combined, the women’s incidence rate is higher in Hillsborough county than in the state of Florida. For men, the incidence rate for all cancer sites combined is higher in Hillsborough. *Among the most significant cancers affecting our region, there is no accepted screening modality for lung cancer [20] and the screening for prostate cancer remains controversial[21]. Therefore, the proposed TB-Navigator Program will initially focus on breast and colorectal cancer.*

B.2 Patient Navigator Program In an effort to close the health disparity gap and to enhance the health experiences of patients, Harold Freeman conceived and initiated the first Patient Navigator Program at Harlem Hospital Center in New York City. Coordination of care was sought out by providing individualized support and assistance in navigating required systems (e.g., financial, health, and social). Key elements of this program include promoting one-on-one contacts to aid patients, families and caregivers to get through a multifaceted and complex health network in a timely manner, providing education strategies for ensuring a better quality of life, linkages to community-based resources, trouble-shooting logistics to, from and within treatment centers, and offering psychosocial support [22-24]. Building on this exemplar model program, we seek to navigate patient lives at the point of a breast and/or colorectal abnormality through timely diagnosis and treatment. Our proposed program intends to offer a unique set of navigation features in collaboration with an inter-disciplinary team at Moffitt and our partnering community agencies, collaborators and community medical stakeholders. Such navigation services will be offered to patients who, irrespective of insurance or income status are at risk to encounter barriers to health care based on a complex interplay of economics, cultural beliefs and institutional barriers. Another key reason for implementing a Patient Navigation Program besides reducing health disparities is health literacy. Patients with low literacy and even those with higher literacy are often overwhelmed by the plethora of cancer-related health information at the point of an abnormal finding. Studies suggest that approximately 90 million adult Americans have insufficient health literacy skills to navigate the current health care system. In the IOM report [25] titled *Health Literacy: A Prescription to End Confusion,* a key recommendation is to develop and test approaches to improve health communication that foster healing relationships across culturally diverse populations (Recommendation 4.2). Further, a key recommendation from the report of the Trans HHS Cancer Health Disparities Progress Review Group[5] is the enhanced attention on the delivery of culturally, linguistically and literacy relevant care. Thus, we believe that the proposed navigator project will be a positive step to generate new knowledge for the advancement of an evidence-based culturally and literacy appropriate Navigation Program designed to repair a –broken- healthcare system and eliminate barriers to cancer diagnosis and treatment.

### **B.3. Community Participatory Research**

Freire’s work in critical pedagogy[26], combined with a critical anthropology of health (CAH) perspective, shapes our approach to the proposed project. These guiding paradigms are complementary in that each acknowledges that structural forces differentially impact health outcomes for disenfranchised populations. However, Freire’s focus is found in educational dimensions of community empowerment. CAH seeks to bring into relief the social, historical and political conditions that produce health and disease, as they relate to local populations. These approaches are consonant with a broader social justice orientation and are useful for deconstructing the sources of health disparities and guiding researchers in fruitful ways for addressing them.

The basic tenets of Freire’s work center on empowerment, the contextualization of peoples’ daily experiences and collaborative, collegial dialogue in adult education [27, 28]. Freire’s work speaks to a variety of action research applications, including those that relate to improving community health of marginalized populations. This guiding theoretical approach articulates exceptionally well with the needs of populations with which we collaborate in our efforts to reduce cancer health disparities in our community. It has long been recognized that factors unexplainable solely by the biomedical model significantly impact the health of a population[29-31]. The 19^th^ century pathologist Rudolph Virchow identified social, cultural, and political factors that were associated with health disparity and disease distribution, including poverty, social class, and famine[32]. Virchow observed that difficult circumstances and deprivations of the working class increase susceptibility to disease as well as to higher mortality rates. He also recognized the political and material circumstances that inhibited disease prevention efforts and viewed advocacy as an essential part of medical research. This early and perceptive understanding of the interaction of biological and social factors that produce illness has had a lasting and significant impact on subsequent critical studies of health and disease in socio-historical context.

Our theoretical perspectives will guide the design of our proposed Navigator Program especially in the developmental phases of the project. Our methodological approach is grounded in community-based participatory research (CBPR), which fosters an environment for the production of collegial and collaborative relationships with community members and academic researchers. Although there is no single definition of community, we believe there are core dimensions that are shared across diverse communities and have internal and external validity[33]. Because the community level is the location of health prevention and health intervention programs, it is significant for obtaining positive health outcomes. According to Flaskerud and Winslow[34], a community health perspective views communities as responsible for the collective well-being and health of their citizens. Meade and Calvo [35] point out that attention must be given to collective rather than individual efforts to ensure that the outcomes reflect the voices of the community and truly make a difference in people’s lives. We also recognize the importance of individual-level empowerment and agency as essential to successful community mobilization. Minkler and Wallerstein’s[36] principles for effective community organization, as well as Labonte’s [37] multilevel empowerment model, allow us to consider both macro level and micro level forces that combine to create both health and disease. In addition, we recognize that all knowledge is produced in a social context and that it is inextricably bound to relations of power. The Freirean emphasis on establishing sustainable, lateral relationships and the explicit recognition of relations of power in the context of joint research efforts are evident in our proposed methods and projects. Further, our conceptualization of the relationship between human agency and health behaviors is congruent both with Freirean ideology and CAH tenets in that we acknowledge that people’s health behaviors are delimited by available resources, yet strength for strategies to succeed in the face of structural constraints is found in community solidarity.

### **B.4. Summary and Significance**

In this section we have presented compelling data that the Tampa Bay region has a significant cancer problem, that the population has richness in racial and ethnic diversity, but also that there are health disparities with regard to the distribution of disease burden and outcome. The existing data suggest that the Patient Navigator Program may effectively contribute to the reduction in health disparities in our region. For this model to be successfully tested and implemented, however, requires an equitable partnership between the only NCI-designated comprehensive cancer center based in Florida, and the community it serves. Our guiding principles for community participatory research were therefore articulated.

# C. PRELIMINARY STUDIES

In order to ensure the successful completion of the proposed study, considerable preparatory work has already been carried out. This has required new expectations from existing partnerships between an NCI -designated comprehensive cancer center and health care providers in the community, the formation of new relationships, and an agreement among all parties on the goals of this collaboration. We have assembled a strong community advisory board that will oversee the development, implementation, and dissemination of results from the study. We have established a strong multidisciplinary team that has the requisite skills and experience to conduct this important work. Therefore, we will begin the preliminary studies section with elaboration of the particular skill sets and expertise of the investigative team. We have worked assiduously for over a decade to develop community outreach and education programs in the region, and these are described. Extensive research has been done to assess the magnitude of the health disparities problem in Tampa Bay. As will be seen, we propose to determine the utility of the Patient Navigator Program on patient satisfaction and quality of life; consequently, we conclude with a synopsis of our work in the measurement and evaluation of these two important constructs.

### **C.1 Investigator expertise and qualifications**

The design and successful completion of the proposed study requires a multidisciplinary team of qualified and experienced investigators. A brief biography of the key personnel is provided below.

*C.1.1 Richard Roetzheim, MD* – Principal Investigator. Dr. Roetzheim is Professor of Family Medicine and Director of Research for the USF Department of Family Medicine. Dr. Roetzheim is a health services researcher who has special interest in primary care, as it impacts cancer outcomes as well as, associated interest in health disparities. Dr. Roetzheim has conducted a number of studies documenting racial and socioeconomic disparities in cancer care and outcomes in the state of Florida[38-40]. Dr. Roetzheim has also conducted studies assessing the effect of primary medical care on cancer outcomes[41-43] and has examined cancer screening among disadvantaged populations[44]. He has also conducted NCI-funded studies to improve cancer screening in primary care settings serving disadvantaged populations[45, 46]. This breadth and depth of experience make him highly qualified to direct the proposed study.

*C.1.2 Cathy Meade, PhD* – Co-Investigator, Dr. Meade is Professor, Department of Interdisciplinary Oncology, University of South Florida, Division of Cancer Prevention and Control. She is also Director of the Cancer Education Program at the H. Lee Moffitt Cancer Center & Research Institute and oversees the cancer center’s education and outreach and continuing education units. She is a nationally recognized expert in the areas of cancer communications, literacy, and cancer education, and is especially skilled in building and sustaining community partnerships and multi-institutional relationships. She is well versed in community-based participatory methods. She is well known for her seminal research in the area of literacy and has produced a broad range of cancer communication materials and media for diverse populations relating to smoking cessation [47, 48]; smoking relapse [49]; colon cancer [50]; prostate cancer [51, 52]; stress and chemotherapy[53]; breast/cervical [54] and stress and radiation[55].

*C.1.3 Paul Jacobsen, PhD* – Co-Investigator, Behavioral Oncologist. Dr. Jacobsen is Professor in the Departments of Psychology and Interdisciplinary Oncology at the University of South Florida. He is also Program Leader of the Health Outcomes and Behavior Program at the Moffitt Cancer Center. Dr. Jacobsen is a nationally recognized expert in behavioral oncology and has extensive experience conducting research on behavioral aspects of cancer detection and control. His work in this area has been funded by the National Cancer Institute and the American Cancer Society. Dr. Jacobsen is currently the principal investigator of an NCI-funded study of quality of life in women undergoing treatment for early stage breast cancer. This study uses many of the same methods proposed in the current study to evaluate quality of life outcomes. Dr. Jacobsen also helped to develop the patient satisfaction evaluation system currently in use at the Moffitt Cancer Center that will be adapted for use in the current study. Dr. Jacobsen will be responsible for the implementation and ongoing supervision of methods used to collect quality of life and patient satisfaction data. He will also participate in the statistical analysis and interpretation of these data in the latter stages of the project.

*C.1.4 Thomas N. Chirikos PhD.* - Co-Investigator/Health Economist. Dr. Chirikos is Professor and Member-in-Residence, H. Lee Moffitt Cancer Center & Research Institute; he is also Professor Emeritus, University of South Florida. An economist by training, Chirikos has conducted research on a wide variety of topics relating to the economics of health and medical care. In recent years, his work has focused on appraising the economic efficiency of cancer diagnosis and treatment. His research has been funded by the NIH, National Science Foundation, the U.S. Department of Labor, and various agencies of the U.S. Department of Health and Human Services, including the Health Care Financing Administration and the Social Security Administration. The results of these efforts have been published in leading journals in the area of health services research. In the proposed project, Dr. Chirikos will assume primary responsibility for conducting the cost-effectiveness analysis of the Navigator intervention.

*C.1.5 Alan Cantor, PhD.* - Co-Investigator. Dr. Cantor is Professor, Department of Interdisciplinary Oncology, University of South Florida, and a member of the Biostatistics Core of the Moffitt Cancer Center. He has over twenty-five years experience as a biostatistician collaborating with medical and epidemiological investigators and conducting independent research in biostatistical methodology. He is author or co-author of over 150 articles in refereed journals as well as two books. Dr. Cantor will receive the study data, convert into a format for analysis, create and perform data checking routines, and produce data summaries and analyses. He has previously played a similar role in an NIH funded screening project that was headed by the Dr. Roetzheim, the P.I. of this proposal.

### **C.2 Health Disparities**

The entire investigative team has an established track record of commitment to addressing health disparities. Dr. Roetzheim has a long-standing interest in whether health disparities in cancer survival are related to screening. His JNCI paper[56] examined the effects of health insurance coverage, race and ethnicity on cancer stage at diagnosis using population-based data for the state of Florida. The hypothesis tested was that patients having HMO insurance would have more favorable stage at diagnosis and that patients lacking health insurance or insured by Medicaid would be diagnosed at later stages relative to patients with commercial indemnity insurance. The report was based on 28,237 Florida residents diagnosed with colorectal, breast, melanoma, or prostate cancers in 1994. Data on insurance payer, comorbidity, and socioeconomic status were obtained through record linkage by means of a probabilistic match using Social Security number, gender, race-ethnicity, and date of birth. The State of Florida, Agency for Health Care Administration (AHCA) maintains both inpatient and outpatient discharge abstracts for admissions to all non-Federal acute care hospitals, all licensed ambulatory surgical centers, all free-standing radiation therapy centers, and all diagnostic imaging centers. The 1990 United States Census was used to obtain data on median household income and median education level (according to race and ethnicity) for each Census tract and ZIP code.

Insurance payer category was significantly associated with stage at diagnosis for each of the four cancer sites examined. Patients insured by Medicaid and those who were uninsured were at greater risk for late-stage disease. Non-Hispanic blacks were at greater risk of late stage diagnosis for breast and prostate cancers. Two payer categories were found to have significantly greater odds of late-stage colorectal cancer than the referent category of commercial indemnity insurance: Medicare HMO and uninsured. Patients who were non-Hispanic black or Hispanic were more likely diagnosed at a late stage independent of their insurance payer. There was a non-significant trend for patients of "other" race-ethnicity to have a more favorable stage at diagnosis. A plausible explanation for a later stage at diagnosis among the uninsured is inadequate access to cancer screening services. Persons lacking health insurance are less likely to receive cancer screening and other preventive services, [17, 57, 58] and may have delayed diagnosis of symptomatic cancers due to inadequate access to health care.

These data motivated Dr. Roetzheim and colleagues to conduct the Cancer SOS project, a four-year cluster-randomized trial of an office systems intervention designed to increase cancer screening in primary care clinics that provide care to underserved populations[45]. Eight clinics were recruited from among community health centers in Hillsborough County, Florida. The study targeted men and women between the ages of 50 and 75 and focused on three cancer screening tests that are widely recommended for this age group. For women, the materials and methods were intended to increase the use of mammograms, Pap smears, and fecal occult blood testing (FOBT). For men, the materials and methods were intended to increase the use of FOBT.

The office systems intervention had two components: a kit of materials used to facilitate screening referrals and a division of office responsibilities and tasks to achieve this goal. The kit of materials consisted of an educational patient brochure, a reminder checklist, and a three-part series of chart stickers, which indicated if specific cancer screening tests were due, ordered, or completed. All intervention materials were available in English and Spanish. The final data set consisted of the combined abstracted records from two independent samples collected at baseline and 12 months post-intervention. Among the three targeted screening outcomes, absolute increases in screening rates were greatest for FOBT screening (14.4% absolute increase in screening rates relative to control clinics). Relative increases in screening rates and numbers needed to treat (NNT) were also more favorable for FOBT compared to other targeted screening tests (Table C.2.1).

Table C.2.1. Effects of the Cancer SOS Intervention

No. Odds Ratio 95% CI P-value NNT

Pap Smears 1057 1.57 0.92 – 2.64 0.096 10.4

Mammograms 1832 1.62 1.07 - 9.78 0.023 14.6

FOBT 1989 2.56 1.65 – 4.01 <0.0001 5.3

This study demonstrated that systematically providing patients relevant information about screening and prompting physician discussions about screening could increase FOBT screening rates in primary care settings. Dr. Roetzheim, in collaboration with Dr. Chirikos, showed that this approach is highly cost effective. *This work underscores our commitment to cancer care for diverse communities and provides a foundation upon which to build the proposed Tampa Bay Patient Navigator Program.*

### **C.3 Community Outreach and Education**

The legislation that created the funding for the H. Lee Moffitt Cancer Center included the mandate that it serve all the residents of Florida, regardless of race, ethnicity, or socioeconomic status. Dr. Cathy Meade directs the Outreach and Education Office, and has established a variety of approaches to accomplish this goal. Several funded educational activities exemplify Dr. Meade’s ongoing focus in the area of cancer communication strategies to reduce health disparities.

*C.3.1 Cancer, Culture, and Literacy. -* In 1998, Dr. Meade initiated the national biennial Cancer, Culture, and Literacy Conferences that aim to improve care across the continuum of cancer control by enhancing the knowledge and skills of professionals responsible for creating multicultural, multilingual, and literacy-sensitive communications. This national meeting, supported by CDC, NCI, CRCHD, Pfizer and the Cancer Research and Prevention Foundation, continues to gain high national recognition as a forum for the exchange of knowledge about the nexus of cancer, culture and literacy and involves extensive interactions with many national scholars and developing investigators. The initial conference spurred the creation of the Cancer, Culture and Literacy Institute, an NCI funded R25 education project (CA 90654). The goals are accomplished through the following educational activities: a) a five-day intensive institute in Tampa, Florida, b) an ongoing Web-based continuing education curriculum, and c) ongoing interactions between scholars/mentors and participants driven by the integration of cancer, culture and literacy themes. To date (Years 01-03), 64 doctoral prepared individuals from a wide array of disciplines (medicine, nursing, psychology, social work, medicine, adult education, nutrition, physical therapy, epidemiology, anthropology, etc.) have participated in this program. Of these 64, approximately 53% were minority (American Indian/Alaska Native 6%, Asian 14%, Hispanic 14%, Native Hawaiian/Pacific Islander 3%, Black/African American 22%, More than one race 3%).

*C.3.2 Multilingual Cancer Education and Outreach for Medically Underserved and Priority Populations -* Dr. Meade has successfully developed a number of programs in this arena. The first targeted Hispanic migrant seasonal farm workers and low income women. Entitled *Por Su Salud*, and funded by an Avon grant, it allowed for the creation and dissemination of culturally appropriate Spanish language materials based on extensive formative research and testing. Each year, a total of 1,000 Hispanic migrant and seasonal farmworkers and low-income rural women are reached with culturally appropriate screening mammography, clinical breast examination and breast health educationand this program was consider one of ten model national programs. The second innovative project, funded by the Cancer Research Foundation of America, was entitled ¡Platiquemos Sobre Su Salud!/Let’s Talk About Your Health! This consisted of the development and evaluation of a Spanish-language breast and cervical cancer educational videotape intervention in terms of knowledge, satisfaction, comfort level, and intentional and actual breast/cervical cancer screening practices among Hispanic migrant and seasonal farm worker women. Reactions to the educational tool were highly favorable and knowledge increased (p<.001). Findings suggested that appropriate educational tools could meet the information needs of at-risk (multicultural, multilingual and low-literacy) populations and contribute to adherence to breast and cervical cancer screening behaviors[59, 60].

*C.3.3. National Work Groups Related to Cancer and Disparities*-Dr. Meade was one of the pioneers who served on the National Cancer Institute’s initial Work Group for Cancer and Literacy (1995-1998) and was a member of NCI’s Comprehensive Working Group on Informed Consent in Cancer Clinical Trials (1996-1998) that addressed cancer information needs of diverse literacy populations and developed policy statements and informed consent guidelines[61]. Most recently, Dr. Meade served as a member of the Institute of Medicine’s Committee on Health Literacy. The outcome of this 18-month activity was the 2004 release of *Health Literacy: A Prescription to End Confusion* in March, 2004. This expertise will be invaluable to the proposed study.

### **C.4 Patient Satisfaction**

Dr. Jacobsen has been involved in numerous studies evaluating quality of life and patient satisfaction in people with cancer. With regard to quality of life, much of this work has focused on women receiving treatment for breast cancer. Early research consisted of cross-sectional studies comparing the quality of life of specific subgroups of breast cancer survivors to the quality of age-matched women with no history of cancer. These studies documented that, relative to women with no history of cancer, quality of life was poorer in women with breast cancer who had previously been treated with standard dose adjuvant chemotherapy[62] or high dose chemotherapy followed by stem cell rescue [63], but not radiotherapy only. [64] Dr. Jacobsen has generated similar findings with regard to the experience of fatigue in these same subgroups of breast cancer survivors. [62-64] In collaboration with Dr. Chirikos, Dr. Jacobsen has also evaluated the relationship of functional impairment to economic well-being in breast cancer survivors. [65] Findings indicated that breast cancer survivors were more likely to be functionally impaired than age-matched women with no history of cancer and that impaired women, in turn, were more likely to reduce work effort and experience downturns in market earning.

With regard to patient satisfaction, work has focused on developing and implementing a cancer-center wide system for assessing patient satisfaction in individuals receiving either inpatient or outpatient care. Beginning in 1999, a group led by Dr. Jacobsen began meeting to solicit input from relevant stakeholders regarding the format and content of a patient satisfaction survey. The group also conducted a review of existing patient satisfaction surveys used with cancer patients. These activities led to the development of a survey that was then pilot tested and finalized. Questions are designed to elicit information about all levels and all aspect of care patients are likely to encounter during either inpatient stays or outpatient visits. This survey is currently administered twice a year to patients randomly selected from among those seen in the outpatient clinics of each of Moffitt Cancer Center’s 14 clinical programs and in each of the center’s 6 inpatient units. Surveys are administered by telephone by trained personnel who enter responses directly into computer files. Over 2,000 patients per year are surveyed using these methods. Summaries of results for specific cancer center units are provided monthly to administrators and faculty members and are then utilized to initiate and evaluate the success of continuous quality improvement projects. Semi-annual reports are used to compare patient satisfaction across clinical programs and across inpatient units. In addition to telephone-administered surveys, focus groups are conducted periodically with patients to explore new areas not addressed in the survey and to allow for more in-depth exploration of relevant issues. Approximately 60 patients per year participate in focus groups. A manuscript describing the development and current operation of this patient satisfaction survey system is currently in press.[66]

### **C.5 Health Choice Network**

Health Choice Network and its member community health centers have extensive experience in serving and working with diverse populations. Located in low-income and predominantly Black and Hispanic communities, most of the Network’s member community health centers have been providing comprehensive primary and preventive health care to racially and ethnically diverse populations for several decades. Overall, 30 percent of community health centers patients in 2001 were Black and 42 percent were Hispanic. Community health centers are required by statute to have Boards of Directors on which at least 50 percent of members are members of the community served and patients. This ensures that the communities served by the centers have a strong voice, and that the centers remain closely tied to the diverse populations they serve. Representation of minorities on the staff and governing Board of Health Choice Network is strong. Of Health Choice Network’s four-member Executive Team, consisting of the President/Chief Executive Officer, two Vice Presidents, and the Chief Financial Officer/Chief Information Officer, one is African American and one is Hispanic. Health Choice Network’s governing Board consists of Chief Executive Officers/Chief Administrators of each member community health center and clinical representatives or Board members from several other member centers. 7 of the 12 members (58%) of Health Choice Network’s Board of Directors are African American and 2 (17%) are Hispanic.

Through centralized program development efforts, Health Choice Network has the capacity to plan, manage, seek grant funding for, evaluate and replicate community-based health promotion and disease prevention initiatives at a higher level than member health Centers would be able to achieve individually. Under the leadership of the Program Development Unit, the Network’s member Centers, along with church leaders in their communities and other partners, have developed and implemented Healthy Body, Healthy Soul, a community-based health promotion and disease prevention program. The mission of the program is to increase access to health care and improve health in predominantly African-American and Hispanic communities through collaboration with churches and other community partners.

The key features of the Healthy Body, Healthy Soul Program include: 1) core partnerships between community health centers and the faith-based community; 2) community capacity building (education and training of staff and partners); 3) the use of Lay Health Educators to conduct effective community screening, outreach, and patient support; 4) access to regular primary and preventive health services; 5) comprehensive disease and case management services; and 6) patient and family education. The core partnership between community health centers and churches is based on the conviction that collaboration between community-based health care providers and the faith community holds great potential as a strategy for conducting effective outreach, pursuing innovative approaches to health education and promotion, and delivering other community-based interventions. As spiritual leaders, clergy members have influence and relationships of trust in their communities, positioning them to reach out to community members effectively with health information that empowers people to make changes in their lives.

Distinctive features of the Program include core partnerships between community health centers and the faith-based community and the use of Lay Health Educators (LHE) to conduct effective community screening, outreach, and patient support. The implementation of the Program occurs on three organizational levels, involving the Network, Centers and faith partners. The Network is the first level of organization and provides overall management, oversight, and evaluation of Healthy Body, Healthy Soul Programs. Centers are the second level of organization and provide primary care, referrals to specialty care, case management, health education and links to social services. Faith partners provide a third level of organization and focus on identifying, screening, and referring community residents to needed health care. They provide the following critical services: 1) cultivating support among other community and faith-based organizations; 2) recruiting and supervising LHE and volunteers; 3) planning/coordinating monthly “Healthy Sunday” events fostering “fellowship of screening” activity; 4) hosting community educational activities; and 5) spiritual counseling. This innovative program will play a key role in the dissemination of the Patient Navigator Program developed and tested in Phase I and II.

### **C.6 The American Cancer Society and Reach to Recovery**

The American Cancer Society (ACS) is a nationwide community-based voluntary health organization dedicated to eliminating cancer as a major health problem by preventing cancer, saving lives, and diminishing suffering from cancer through research, education, advocacy, and service. With more than two million volunteers nationwide, the American Cancer Society is one of the oldest and largest voluntary health agencies in the United States. The Florida Division has more than thirty offices to serve local communities, and 80,000 volunteers directing the Society’s programs at the local level. These dedicated volunteers donate their time and talents to further cancer research; educate the public about early detection and prevention; advocate for responsible cancer legislation in the local, state, and federal governments; and serve cancer patients and their families to help make the cancer experience a little easier.

In the ACS Reach to Recovery Program, breast cancer survivors provide one-on-one support and information to help individuals cope with breast cancer. Specially trained survivors serve as volunteers, responding in person or by phone to the concerns of people facing breast cancer diagnosis, treatment, recurrence or recovery. There are currently over 60 Reach to Recovery volunteers in the Tampa Bay area. Our design will capitalize on this existing resource of trained volunteers to augment the Navigator Program.

# D. MATERIALS AND METHODS

The overall research design of the Patient Navigator Program is presented in figure1 (D.1). We follow with a description of the study population (D.2) and an overview of Phases I (D.3), II (D.4) and III (D.5). Finally, we end with the timeline for the proposed project (D.6).

**D.1 Overview**

**
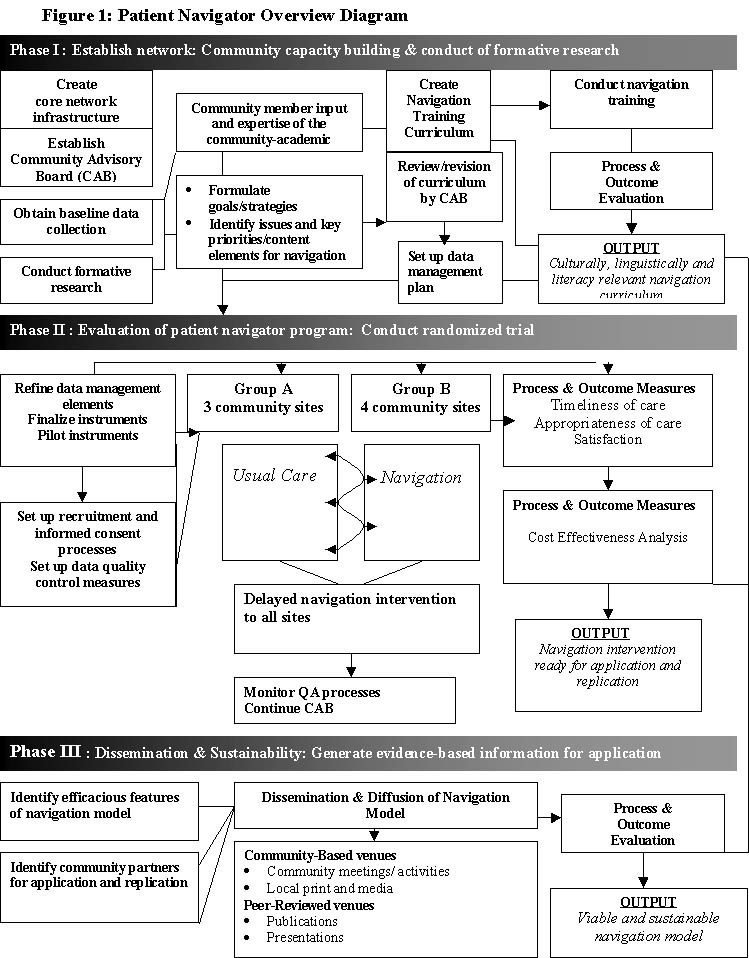
**

**D.2. Study Population and Inclusion/Exclusion Criteria**

Hillsborough County, the fourth most populous county in Florida (population 1,027,318), encompasses metropolitan Tampa, its surrounding suburbs, and primarily rural areas to the west and south. Hillsborough County has a diverse population including populations noted for disparities in cancer care and outcomes (Hispanics, African Americans, patients residing in rural settings).

This project will originally target clinics that specifically provide primary care to disadvantaged populations within Hillsborough County. Two of these clinics (Tampa General Health Clinics) are located in inner city Tampa, serving primarily minority and lower socioeconomic populations. Three clinics (Suncoast Community Health Centers) are located in primarily rural areas of Hillsborough County, for which Hispanic patients and migrant farm workers are important populations served.

Each of these clinics has previously participated in an NCI-funded intervention (Cancer SOS Project, summarized in C.2) to promote cancer screening in primary care clinics serving disadvantaged populations. The investigative team has an excellent working relationship with clinic administrators and primary care providers. The SOS project also led to a better understanding of the relevant barriers to screening and appropriate follow-up care, which contributes to the feasibility of the proposed study. A brief description of the clinics, and a summary table of their key characteristics are as follows.

*Tampa General Healthcare (TGH)-* Tampa General Hospital is a private not-for-profit corporation governed by a volunteer Board of Directors. As a teaching facility affiliated with the University of South Florida College of Medicine, Tampa General partners with academic and community institutions to support both their teaching and research missions. As the region’s leading safety net hospital, TGH is committed to providing high quality health services to all residents. TGH operates five ambulatory care clinics, two of which are located in inner city Tampa.

*Suncoast Community Health Centers -* Three clinics serve the Suncoast Community Health Center, Inc. network. The clinics are located in three cities each located approximately 20 miles outside the Tampa Metropolitan Area.

| Table D.2. Characteristics of participating community health centers in Tampa region | | | | | | |
| --- | --- | --- | --- | --- | --- | --- |
| Clinic Name | Screening Visits  # per year | African American  (%) | Hispanic  (%) | Under-insured  (%) | Low literacy  (%) | Spanish speaking  (%) |
| Ruskin | 33,800 | 5 | 50 | 85 | 40 | 30 |
| Plant City | 26,000 | 30 | 40 | 85 | 65 | 45 |
| Dover | 36,400 | 0 | 75 | 100 | 50 < 6^th^ gr | 75 |
|  |  |  |  |  |  |  |
|  |  |  |  |  |  |  |
| Health Park | 20,800 | 40 | 20 | 70 | 40 | 5 |
| Midtown | 19,500 | 35 | 35 | 85 | 30 | 5 |

Since the start of the project, the number of participating clinics has increased (n=12) and they have been randomized as described below. Note that randomization occurred within sponsoring organization and for organizations with an odd number of clinics randomization was performed to insure that there were a greater number of intervention clinics than control.

**Tampa General Health Care**

TGH Family Care Center Healthpark

(30^th^ Street) Intervention

TGH Family Care Center Kennedy Control

**Suncoast Community Health Centers**

Ruskin Health Center Intervention

Plant City Family Care Intervention

Dover Health Center Control

**Premier Community Health Centers**

Dade City Family Health Center Intervention

Zephyrhills Family Health Center Control

**Manatee Community Health Centers**

Southeast Family Healthcare Center Intervention

Parrish Family Healthcare Center Intervention

North Manatee Family Health Center Control

**Pinellas County Community Health Centers**

Johnnie Ruth Clarke Health Center Control

Community Health Centers Pinellas Park Intervention

Data to be collected about participating clinics:

Each clinic will be sequentially assigned a unique 2 digit ID number which will be reported in Table 1B provided to NOVA (see data dictionary).

Standard information will be collected from each clinic at the start of their participation in the project and again at the conclusion of the project. The information to be collected is described in Table 6 of the data dictionary.

D.2.1. Inclusion/Exclusion Criteria

General exclusion criteria include the following:

- Cognitively impaired
- Institutionalized
- Persons under age 18
- Previous navigation for cancer (either in PNRP or elsewhere)
- Known to be pregnant at enrollment
- Previously treated cancer (other than non-melanoma skin cancer) less than five years from diagnosis

Patients will be eligible for inclusion in the study if they receive primary medical care at one of the participating medical clinics and are noted to have a potentially cancer-related abnormality as defined below. Patients must be at least 18 years of age, but there will be no exclusion on race or income. Patients who are newly diagnosed with breast or colorectal cancer and who have not undergone any treatment will be eligible for the study.

*D2.1.1 Breast Cancer-related Abnormalities*.-- Patients having a breast-related abnormality that in the opinion of the primary physician warrants further evaluation will be eligible for inclusion. Subjects having the following breast related abnormalities will be eligible for inclusion:

- Health care provider confirmed breast mass or other abnormality suspicious for cancer prompting referral to specialist or referral for further testing
- Abnormal or incomplete mammogram (BIRADS 0, 3, 4, 5)
- Ultrasound abnormality (indeterminate or suspicious solid lesion, complex cyst, BIRADS 0,3,4,5)
- MRI abnormality (BIRADS 0,3,4,5)
- Pathologically confirmed DCIS or invasive cancer that has not been treated

Patients having symptoms potentially related to breast cancer (abnormality noted on self-exam, breast pain, nipple discharge) will also be eligible if there are corresponding suspicious findings on clinical examination warranting referral. Patients having breast abnormalities that are fully diagnosed and treated within the primary care setting, without the need for additional evaluation, will not be eligible for inclusion in the study.

*D.2.1.2 Colorectal Cancer-related Abnormalities*.-- Patients having an abnormality potentially related to colorectal cancer, and requiring further evaluation, will be eligible for inclusion. Conditions meeting eligibility criteria include:

- Rectal bleeding in person 30 years or older with referral to specialist
- Suspicious finding on rectal exam (mass) with referral
- Abnormality on sigmoidoscopy (adenomatous/malignant polyp, mass, invasive cancer)
- Abnormal colorectal imaging (space occupying lesion by ACBE, virtual CT colonography).
- History of malignant polyp
- Positive FOBT / Guaic with referral to specialist in persons 30 years and older
- Newly diagnosed and pathologically confirmed colorectal cancer prior to treatment
- Abnormality on colonoscopy requiring additional evaluation (mass, polyp requiring excision, incomplete or unsatisfactory examination)

Patients with abnormalities that are fully diagnosed and treated in the primary care setting, without the need for additional evaluation, will not be eligible for inclusion in the study as they would have no need for navigation.

**D.3. Phase 1: To work closely with our community partners to conduct a formative evaluation of the acceptability, appeal, and appropriateness of a Patient Navigator Program.**

###### **D.3.1 Community Advisory Committee**

A multi-disciplinary and multi-cultural advisory committee has been formed to ensure the cultural sensitivity, appropriateness, and effective implementation of the study as well as to oversee the dissemination and translation of findings. This Committee is critical to the success of the project, given the focus on testing the Navigator Program within a community network of healthcare providers. The Committee includes representatives of partner institutions, as well as key personnel at Moffitt or the University of South Florida. In particular, the Committee will include Reverend Ted Grier, MHA, Director of Faith Based and Community Initiatives with HealthChoice Network; LaWanda Courtney, the Manager of the Office of Institutional Diversity at Moffitt; Rony Francois, M.D., MSPH, Ph.D. a professor at the USF College of Public Health; Susan Rabel, Vice President, Cancer Control, American Cancer Society, Florida Division; Brantz Rosell, CEO for Suncoast Community Health Center; Dianna Lopez, Suncoast Community Health Center; Charles Bottom, CEO, Tampa Community Health Center; Gloria Elliott, Tampa Community Health Center; Okey Ryan, CEO, Pinellas County Community Health Center; and Wanda McGinnis, a Patient Advocate.

The Committee will meet two times in Year 1. At the first meeting their role will be explained, and modified as necessary. The goals of the study will be restated and the timeline for each of the phases will be reviewed. Input will be solicited on the composition of the focus groups (see D.3.2) and barriers to abstraction of baseline data will be addressed (see D.3.3). The second meeting in Year 1 will be dedicated primarily for evaluative input on the results of the focus group, the training plans for the Navigators (see D.3.4), and implementation issues related to recruitment. The Committee will then meet once per year through year 4 to monitor progress, and contribute to solutions for covering medical care costs for uninsured. In Year 5 the Committee will meet twice to assist in the finalization of the dissemination plan.

**D.3.2 Multi-step Systematic Process Leading to Navigation Training Program**

Emphasizing the collective knowledge and experience of our team, we aim to implement a multi-step process to create a unique Navigation Program based on a needs assessment. Methodologies used to develop the curriculum reflect the approaches outlined in the NCI’s Stages in Health Communication Model which offers the opportunity for continuous assessment, feedback, and improvement and is a helpful and sequential framework to hold ideas together and triage major concepts in a sensible fashion[67]. Development of the Navigation Program will begin with the conduct of focus groups in Phase I among cancer patients and key community informants that will contribute to programmatic goals and objectives and more finely focus key areas for navigation. Construction of the content domains of the proposed Navigation Program will be guided primarily by the focus groups, draw on our baseline cancer data, national and local cancer statistics and literature on existing navigation programs. We plan to fully develop the curriculum in Year 1 in close collaboration with our community members, community advisory board, and collaborators through a series of iterative steps (See Figure D.3.2, schema).

## Figure D.3.2.1 Overview of Navigator Training Module Development

## Multi-step systematic process

**Preliminary navigation training**

**module**

**Modify**

**&**

**Revise**

**Modify**

**&**

**Revise**

Research team meets review questionnaire, debrief and provide feedback and continuously revise training module

Focus Group Findings

Experience and expertise of community and research team

Review literature

*Create an inventory of possible content areas*

- *Peer linkages*
- *Social service resources*
- *Financial resources*
- *Communications/Interactions*
- *Impact of culture and literacy on services*
- *Use of forms*

*Using outline/ flowcharts of domains; Create training module*

**Iteration 1: Review by Community Advisory Board**

**Iteration 2: Review by community stakeholders and consultants**

Key emphasis on cultural acceptability and understandability

### **Part A**

**Development**

**Part B**

**Refinement**

Review by CAB

**Iteration 3: Final Review and Revisions**

**Solid Navigation Training Module set for implementation**

*D.3.2.1 Components of the Navigation Program*- A key aspect of the proposed navigation program is the delivery of culturally competent services. Meleis[68] cogently describes culturally competent care as that care which exhibits sensitivity to the differences in individuals based on their vast experiences and responses due to their backgrounds, sexual orientation, socioeconomic status, ethnicity and cultural background. She also depicts several properties that make up the “essence of health professionals” who deliver culturally competent care. First, they possess an explanatory system that values diversity. This is a system that is not drained by the constant attempt to interpret symbols but rather is energized by the variations. Second, they show expert assessment skills to discern different and similar patterns of responses that help to plan appropriate educational interventions. Third, culturally competent professionals are aware of the diversity of communication patterns and how language and communication influence “trust within the relationship.” Culturally competent professionals also recognize how marginalization may increase health risks for individuals and that using the expertise of insiders in the culture is a highly valued skill. Last, the professional who delivers culturally competent care readily acknowledges differences and does not tolerate inequities. Thus, the provision of culturally competent care and expert communications are at the heart of our proposed Navigator Program. Besides *cultural competency and responsiveness* as the cornerstone features of our proposed Navigation Program, other critical tenets include *health outreach and education*, the *appropriate use of health data tools* and *linkages to key community and healthcare resources*. It is only through effective, relevant and customized outreach and education strategies that we can enable patients and their families to become equal and empowered partners in their health care. Also, the establishment of linguistically and relevant data tools are essential for health program planning, development, and evaluation in order to track, respond, and meet the on-going, changing, complex, multi-faceted components of the health care system. We are aware that if funded, that we will collaborate with the CRCHD and share a uniform protocol and standardized data collection and documentation procedures. While we are unsure of the complete topics to be covered in the training, we anticipate that some of the following subjects will be included based on our experiences.

Table D.3.2. Draft Key Topical Components of the Training Program

| Training expectations  Cancer and minorities  Barriers to health care  Navigation and outreach services  Patient advocacy  Setting goals and objectives  Creating and establishing your role  Things to know when working in the community  Cultural and literacy considerations  Communication skills  Psychosocial skills | Literacy  Defining existing resources for financial assistance/services  Coordination of patient services  Finding, building and securing community resources  Information gathering techniques  Facilitating access and information to patients  Documentation of navigation encounters and data collection  Strategies to overcome potential navigation challenges  Dealing with conflicts and decision-making processes  Using training participants’ experiences as resources  Stories from the field |
| --- | --- |

Our program will include navigators to bridge the gap in provision of timely and quality health care for underserved groups within our community who have an abnormal breast or colorectal screening finding or a diagnosis of breast or colon cancer. We intend to base our cadre of navigators at community centers whereby they will be more entrenched into the work milieu within the community center. Also, we plan to have a lead navigator who will offer day-day team leadership and availability with the community-based navigators. All navigators will work closely under the oversight and guidance of Dr. Cathy Meade and Ms. Marlene Rivera, who bring to this project extensive community-based nursing experience, and background in sustaining community-based partnerships[35, 54, 59]. We are also most fortunate to have as part of our team, *Ms. Leka Murdock,* who worked as the first patient navigator in the Harlem program. Her practical and extensive experiences and ideas will greatly complement our proposed training program and serve to infuse a *worldview* perspective of issues for successful navigation outputs.

*D.3.2.2 Focus groups* The use of focus groups will augment findings obtained from the literature about key Navigation Program components, but from a *local* perspective. It is expected that such insights (from patients and community key informants) will further define key navigation issues within our community. We plan to conduct a total of 7 focus groups (4 patient; 3 community informant). Dr. Meade brings experience in the conduct of focus groups and will lead this aspect of the study. To supplement focus group data, we will conduct in-depth interviews with up to 30 navigated and non navigated (from the community) patients to help assess patients’ perspectives regarding whether patient navigation was useful in overcoming personal, logistical, and health system barriers to care.

##### *D.3.2.3 Overall Design and Background* Focus groups are a qualitative data gathering technique that is sensitive to the participant’s experiences, perceptions, and beliefs and elucidates the cultural context for a wide range of perceptions and attitudes relating to a defined topic[69-72]. The primary goal of the focus group methodology used in this study is to obtain information that will be useful in the design of a culturally sensitive, literacy appropriate and socially relevant Patient Navigation Program for implementation in community-based centers as well as to further help define issues important to our overall project. Specific focus group objectives are:

- To identify patient experiences when accessing the health care system for cancer care (e.g., social, personal, environmental, financial, literacy and cultural barriers)
- To obtain insights about relevant content domains for inclusion in the patient navigation module.
- To examine pertinent community issues important to the development of the overall Navigator Program design.

*D.3.2.4 Sample and Recruitment Techniques* A focus group should be small in order for discussion to flow more easily and allow the participants to express their views [73]. Composition ideally should vary between six to twelve members [74]. We will recruit for 12 possible participants per focus group, expecting a slightly lower number of participation based upon our previous experience in conducting focus groups within the community. Focus group locations and promotions will be facilitated through our established extensive network of community partners known to serve low-income residents. In planning focus groups, they should be homogeneous with regard to certain characteristics relating to the purpose of the research[69, 73, 74]; . Gender is a variable known to influence people’s communication patterns and how they access information and communicate in terms of vocabulary[75]. Thus, we will stratify our patient focus groups by gender and they will represent patients who have received treatment for breast and/or colon cancer within the past one year. We will also conduct three focus groups among key community informants (e.g., community-based providers, medical stakeholders, outreach workers) known to provide care for the medically underserved (Figure 2).

**Figure D.3.2.2. Overview of Focus Group Methodology**

**Total 4 Groups**

**Total 3 Groups**

Type Group

Gender:

Age

Group:

A. Community Stakeholders

B. Medical Providers

C. Outreach workers

Patients

N=1

**N=10**

Women

**N=10**

**N=10**

**N=10**

Men

B

. Outreach workers

C

. Outreach workers

A

. Outreach workers

**N=10**

. Outreach workers

**N=10**

. Outreach workers

**N=10**

. Outreach workers

##### *D.3.2.5 Procedures* The methods and procedures used to conduct focus groups will be based on standardized methods[76, 77]; . Although we plan to conduct a total of 7 focus groups in Phase I, it is important to maintain flexibility in the design of such formative research. For example, the final number of groups will be based on the principle of theoretical saturation [77]. If we find that we are receiving inconsistent information from the focus groups, we will conduct additional groups until no new information is obtained.

*D.3.2.6 Focus Group Analysis Plan* Focus groups will be tape-recorded, transcribed verbatim, manually coded and then reviewed by members of the research team following the approaches used by Morgan [77]. Data will be analyzed using a content analysis technique, which identifies emergent themes, or trends, from the focus group transcripts. Analysis of focus group data includes a “mechanical” stage (organizing and dividing the data into a useful scheme), and an “interpretive” stage (identifying criteria for organizing the thematic codes; [78]. All documents, transcripts and tapes will be stored in a locked file cabinet in the Cancer Control Education Research Department at the Moffitt Cancer Center.

### **D.3.3 Baseline Data Collection**

We will collect baseline data from all participating clinics. Subjects will be eligible for medical record abstraction if they were seen at one of the participating clinics within one year of the clinic’s start in the project and if the subject has a breast or colorectal screening abnormality that meets eligibility criteria (see above). Specific data elements that will be collected are listed in the NOVA data dictionary (Breast - Table 7, Colorectal -Table 9).

Questions to be addressed using baseline data include:

*1. What is the average length of time from abnormal screening test to definitive diagnosis?*

*2. Are there any predictors of timeliness of diagnosis?*

For patients diagnosed with cancer, the following additional questions will be addressed.

*1. How many breast and colorectal cancers are diagnosed each year?*

*2. At what stage are these cancers diagnosed?*

*3. What is the average length of time from abnormal screening test to definitive diagnosis?*

*4. What is the average length of time from diagnosis to initiation of definitive treatment?*

*5. For each treatment modality (surgery, radiation therapy, chemotherapy), what percentage of patients is recommended to have treatment and what percent complete this therapy?*

*6. What physicians/ facilities currently provide diagnostic and cancer care?*

Baseline data will be helpful in establishing baseline estimates of project outcomes, confirming sample size requirements, and suggesting navigation strategies. Chart abstractions will be conducted on the clinic premises and no information that could uniquely identify a patient will be recorded. Thus the final dataset will be de-identified for human subjects purposes.

*D.3.3.1 Medical Record Abstraction* We will follow established guidelines and recommendations to insure complete and accurate chart abstractions[79-81]. All parts of the medical record will be reviewed including preventive medicine flow sheets (if present), progress notes, laboratory reports, and records from other providers. Chart reviews will be conducted by trained medical record abstractors. Training manuals for chart abstraction will be developed along with standardized training examples. To the fullest extent possible, chart abstractors will be blinded to the study hypothesis. To ensure reliability of chart reviews, the project director will re-abstract 20 charts for each medical record abstractor and calculate values of kappa for key variables. Values of kappa > 0.80 will be considered adequate evidence of reliability. Throughout the project the director will also similarly perform spot checks of medical record abstractor reliability. If values of kappa fall below this threshold corrective action and retraining will occur. Medical record abstractions will be conducted at primary care clinics, with additional abstractions of records obtained by all diagnostic, consulting, and treating facilities. Baseline data will be extracted for one year prior to clinic randomization to intervention arm.

Data will be entered on site using MedQuest, a software system for chart abstractions that is available free from the Centers for Medicare and Medicaid Services (<http://www.cms.hhs.gov/medquest>). The software system includes the ability to create data dictionaries and data entry rules, including enable/disable variables, enable/disable screens, validation rules, exit case rules, skip to, and save to rules. The rules provide the ability to restrict, enforce and validate the data that are being entered. Help screens are available through pop up windows. The program also allows data quality assessments, including comparisons between abstractors, and has data importing and exporting features. The variables to be abstracted are summarized in the following table.

| **Clinical data regarding abnormality** | ***Data Source*** |
| --- | --- |
| Initial abnormality – description (ex. Breast lump, abnormal mammogram) and date of onset | *Clinic record* |
| Date abnormality first addressed by primary provider | *Clinic record* |
| Diagnostic procedures performed (dates, results) | *Clinic record, outside records* |
| Physician referrals (type, dates referred, dates completed) | *Clinic record* |
| Date definitive diagnosis given to abnormality | *Clinic record, outside records* |
| **Clinical data on cancer diagnosis and treatment** | **Data Source** |
| Type of cancer, AJCC Stage at diagnosis | *Clinic record, outside records* |
| Treatments initially recommended | *Clinic record, outside records* |
| Referrals for treatment (type, dates referred, dates referral completed) | *Clinic record, outside records* |
| Cancer directed surgery (type, dates) | *Clinic record, outside records* |
| Cancer directed radiation therapy (type, dates initiated, dates completed) | *Clinic record, outside records* |
| Cancer directed chemotherapy (type, dates initiated, dates completed) | *Clinic record, outside records* |
| Cancer directed hormonal therapy (type, dates initiated) | *Clinic record, outside records* |
| Palliative / supportive therapies (types, dates initiated) | *Clinic record, outside records* |
| Hospitalizations (dates, length of stay) | *Clinic record, outside records* |
| Vital status / *Date of death (if applicable)* | *Clinic record, outside records* |

| **Other relevant patient medical information** | **Data Source** |
| --- | --- |
| Patient age, gender, race-ethnicity, marital status | Clinic record, Intake interview |
| Patient employment | Clinic record, Intake interview |
| Patient educational attainment | Intake interview |
| Type of residence (home, apartment) | Intake interview |
| Who else lives with patient | Intake interview |
| Health insurance type | Clinic record, intake interview |
| Family history of breast or colorectal cancer | Clinic record |
| Estrogen replacement therapy | Clinic record |
| History of benign breast disease (previous biopsies) | Clinic record |
| Comorbidity (Charlson Comorbidity Index) | Clinic record |
| Number of health care visits since index visit | Clinic record |
| Previous mammograms (dates, results) | Clinic record |
| Previous clinical breast examinations (dates, results) | Clinic record |
| Previous colorectal cancer screening tests (types, dates, results) | Clinic record |

### **D.3.4 Recruitment and Training of Navigators**

Patient navigators will be recruited through the community-based centers and our community partners. Personal characteristics important when hiring navigators and consistent with the Freeman model[22] include: being culturally attuned to the community; knowledgeable about community systems and resources; having strong problem-solving abilities; being able to connect with required systems that impact funding sources; and being cultural competent (See Appendix for proposed job description). The patient navigators will undergo extensive training under the guidance of an interdisciplinary academic-community team including Dr. Meade, Dr. Jacobsen, Ms. Rivera, Ms. Murdock and our community partners (Suncoast, Tampa Community Health Center and Health Choice) in Phase I. We will provide an initial three-day training seminar for newly recruited navigators from our partner community-based health and social service agencies to enhance the skills and capacity to effectively implement the Navigation Program. The three-day training program will include didactic, group, role-playing and actual fieldwork, and be based on adult learning principles. This training will include instruction in how to implement the navigator protocol with patients as well as instruction in how to interact with health care professionals to promote delivery of timely high quality care to patients being navigated. Navigators will also receive information about resources available to help patients with regard to practical, financial, and emotional concerns. Case studies will be reviewed and problem-solving and skill-building techniques in navigation will be emphasized. Critical to this aspect of the training is understanding linkages to key medical stakeholders and processes to achieve successful navigation. As part of the training, each navigator will be audiotaped delivering key elements of the intervention to a limited number of patients not participating in the randomized trial. Audiotapes will be reviewed by Dr. Meade and her team who will provide feedback individually to each navigator. During the course of the study, 20% of each navigator’s initial and follow-up contacts with participants will be recorded. The audiotapes will be reviewed by Dr. Meade and Ms. Rivera who will provide feedback to each navigator about any possible drift from the original intervention protocols while recognizing the need for customized and personalized care. Feedback will be provided during regularly scheduled meetings, (biweekly) at which time Ms. Rivera and the lead navigator will review with each navigator any issues and problems that may be occurring in the delivery of the intervention. Bi-weekly meetings will be held with the navigators to boost skills, camradarie and encourage problem-solving skills.

**D.4 Phase II: To conduct a group randomized controlled trial to evaluate the Patient Navigator Program.**

### **D.4.1 Overview of Methods**

The goal of Phase II is to test whether a Patient Navigator Program implemented in the Tampa Bay region will lead to improved timeliness of cancer care, appropriateness of cancer care, and patient satisfaction. After careful consideration of several study designs for scientific rigor, feasibility, and acceptability by our community, we have selected a group randomized design (D.4.2), with the “group” being a community health center. The intervention arm will receive the Navigator Program first, and the control arm will receive the intervention, but delayed in time to permit the evaluation of program effectiveness. Patients within groups will be recruited by Clinical Research Assistants at the time of a suspicious screening result for cancer of the breast or colorectum (D.4.3). The Patient Navigators will assist the patients who have abnormal screening findings obtain timely appointments, referrals, and financial support that may be necessary. Data will be collected from medical records regarding timing of care and adherence of care to NCCN guidelines [82, 83] with chart abstractions occurring at 12 months, and patient satisfaction occurring for a randomly chosen subsample at baseline and again at 3 months. Data will be collected to assess, among other things, the patient’s navigation through, and satisfaction with, the health care delivery system (D.4.4).

### **D.4.2 Group Randomized Design**

Five clinics will initially be participating in the randomized evaluation of the Navigator intervention: three funded by the Suncoast Community Health Center, Inc, and two by Tampa General Healthcare. Three of the five will be assigned to the initial Navigator intervention, while the other two will receive this intervention later after the evaluation is complete. In order to assure that each participating organization can feel that its patients are benefiting from the organization’s participation in this project, the randomization will assure that at least one clinic affiliated with each organization is chosen for the immediate intervention. Thus we will design the randomization schema to assure that two of the three Suncoast clinics, and one of the two Tampa General clinics receive the immediate Navigator intervention.

Seven additional clinics have been recruited to the study and have been randomized as described above.

###### **D.4.3 Patient Recruitment**

As described previously, seven clinics will provide the Navigator Program and five clinics will not provide the Navigator Program based on random assignment. A description of the Navigator Program to be provided at those clinics randomized to this condition follows. We will make no attempt to alter the delivery of services at those clinics not randomized to deliver the Navigator Program. Rather, we will use both observational and survey methods to characterize the processes that typically occur in these clinics once patients are identified as having a breast or colorectal abnormality. These data will be used to characterize the extent to which delivery of care differed in our two intervention conditions.

The eligibility criteria were defined in D.2.1 above. Subjects having cancer-related abnormalities will be identified by research assistants using a variety of methods. Methodologies for identifying patients will be refined and pilot tested in the project’s first year. Procedures for subject identification and recruitment will be identical for intervention and control clinics. Once identified, patients will be contacted by research assistants for recruitment into the study. Research assistants will use the following sources to identify patients:

*Appointment records*: Appointment records provide the reason for each patient’s visit and will be assessed weekly by research assistants. This information will be used to identify cancer related symptoms (i.e. breast lumps, rectal bleeding).

*Screening logs*: Participating clinics maintain screening logs to document completion of cancer screening tests such as Pap smears, mammograms, and FOBT. Research assistants will track screening logs on a weekly basis to identify patients having abnormal results on mammography or FOBT.

*Referral coordinators*: Research assistants will also make at least weekly contact with referral coordinators at each clinic to identify patients who may have been referred for cancer related problems.

A research assistant assigned to each participating clinic will review medical records in order to identify potential participants who meet these eligibility criteria. Patients found to meet the eligibility criteria will be approached by the research assistant who will first provide a brief verbal explanation of the study. Patients wishing to receive more information will then be provided with a copy of the informed consent form to review. The research assistant will remain available to answer any questions. Those patients wishing to participate will then be asked to sign the informed consent form. Participants will then undergo study procedures as described below.

*Lifetime Cancer Screening*: We will also identify eligible subjects by reviewing results of mammography screening services provided by Lifetime Cancer Screening, either on site or through mobile screening vans. Subjects meeting eligibility criteria will be recruited into the navigation arm if they are patients of intervention clinics or recruited into the control arm of the study if patients of control clinics.

**D.4.4 Description of the Navigation Program**

The goal of the Navigator Program is to facilitate timely access to needed patient care interventions for patients exhibiting abnormal findings related to a breast or with a colorectal cancer screening abnormality. The principles that drive an effective navigation plan of intervention are based on patient/family assessment of needs, development and implementation of a plan of action and evaluation of outcomes. Additionally, a well- integrated Navigation Program with our partner agencies is needed to assure a seamless, well-coordinated program is established where the full scope of resources within the agencies, the cancer center and the community are utilized to meet patient needs. We envision that the navigator assigned at the community health center level will be well integrated into the existing site systems and well acquainted with community resources. The navigator will collaborate on a day-to-day basis with the health center interdisciplinary team, including health center outreach workers, financial advisors and clinical staff performing case management services.

Once a patient is enrolled in the study at a clinic randomized to provide the Navigator Program, the following activities become activated as part of the processes used to navigate the patient into and through the system. Simply put, the navigator helps the patient become informed about the various services, coordinates the care and assists them with decision-making tools. Key to the success of the navigator role is awareness of the availability of resources and creating meaningful and timely linkages. This role involves coordination of services, education and support offered in a highly personal and customized manner to help patients and families through this journey. The navigator provides an important access point to a wide array of services in a coordinated fashion that reduces the ‘shopping’ for providers and services. Specific tasks to be taken on by the patient navigator include the following:

- Effectively utilizes patient assessment tool to identify barriers to timely health services including transportation, health insurance funding, psycho-social needs, health education needs, child care needs, cultural or belief system barriers, unemployment, etc.
- Develops and implements intervention plan to assist patients and families in accessing, seamless, coordinated care based on identified needs, e.g. timely appointments, transportation services, referral to social services, etc.
- Participates with the primary care and cancer team.
- Follows up with patients who missed appointments to assure all barriers to care are addressed as needed.
- Accompanies patient and family to appointments when necessary, serving as a “cultural translator” and patient advocate when appropriate.
- Serves as a liaison between the community and cancer centers/specialists.
- Provides emotional and spiritual support as needed to patient and family.
- Provides (makes) appropriate referrals and linkages to cancer center and community resources; assists patients with securing financial benefits and entitlements.
- Utilizes interventions and strategies that are appropriate to the population, i.e., considering culture, literacy, language, age and gender.
- Evaluates interventions and outcomes continuously and then reformulates plan of action based on responses and needs.

While protocols will be established to guide the navigator in this systems role, there is also a keen focus on client-centered care. In summary, the navigator serves as a constant, that is, a person who consistently guides the patient through the system. The navigator is someone who can piece together complex information in logical and easy-to-understand ways to achieve timely and appropriate access to vital cancer care.

### **D.4.5 Data Collection**

**Data accrual**

We will collect information on the number of subjects who are referred into the study, the number who are successfully consented, and the reasons that referred subjects are not enrolled (couldn’t be contacted, not eligible, declined, etc.).

**Navigated subjects**

The following data will be collected on navigated patients.

*Demographic Survey*

Navigated patients will provide information on demographic characteristics that are unlikely to be found in the medical record. This information will be collected in person by the navigator at the time subjects provide consent. See Demographic Survey for description of individual items.

*Measures of health literacy*

We will administer the REALM (long version) to English speaking subjects at baseline as our standard measure of health literacy. For Spanish speaking subjects, we will administer the 50 item SAHLSA instrument, also at baseline.

Measures of depression

As a potential barrier to receiving cancer related health care, participants will be screened for depression using the PRIME-MD Patient Health Questionnaire (see attached copy of instrument).

*Additional instruments*

For subjects with non cancer diagnoses, the following instruments will be administered within three months of diagnostic resolution: **Impact of Events Scale**, **Communication and Attitudinal Self-Efficacy** (CASE) general scale, **satisfaction with care survey**, **satisfaction with navigator survey**. These instruments and instructions for how they are administered are found in the NOVA Data Dictionary.

For navigated subjects with a diagnosis of cancer, the following instruments will be administered three months after initiation of treatment; Impact of Events Scale, Communication and Attitudinal Self-Efficacy (CASE) cancer scale, satisfaction with care survey, satisfaction with navigator survey.

Chart abstractions will be performed on the medical records of navigated patients approximately three months after diagnostic resolution (three months after initiation of treatment for cancer patients). Information that will be abstracted is described in the NOVA Data Dictionary

Patient demographics Table 2

Patient / Family Medical History Table 3

Charlson Comorbidity Table 4

Breast related outcomes Table 7

Colorectal related outcomes Table 9

For persons diagnosed with cancer, we will also obtain data from the treating hospital tumor registry if possible.

**Control subjects**

The following data will be collected on control subjects.

*Demographic Survey*

Control patients will provide information on demographic characteristics that are unlikely to be found in the medical record. This information will be collected in person by the research assistant at the time subjects provide consent. See **Demographic Survey** for description of individual items.

*Measures of health literacy*

We will administer the REALM (long version) to English speaking subjects at baseline as our standard measure of health literacy. For Spanish speaking subjects, we will administer the 50 item SAHLSA instrument, also at baseline.

*Additional instruments*

For subjects with non cancer diagnoses, the following instruments will be administered within three months of diagnostic resolution: **Impact of Events Scale**, **Communication and Attitudinal Self-Efficacy** (CASE) general scale, **satisfaction with care survey**, **satisfaction with navigator survey**. These instruments and instructions for how they are administered are found in the NOVA Data Dictionary.

For control subjects with a diagnosis of cancer, the following instruments will be administered three months after initiation of treatment; Impact of Events Scale, Communication and Attitudinal Self-Efficacy (CASE) cancer scale, satisfaction with care survey, satisfaction with navigator survey.

Chart abstractions will be performed on the medical records of control patients approximately three months after diagnostic resolution (three months after initiation of treatment for cancer patients). Information that will be abstracted is described in the NOVA Data Dictionary

Patient demographics Table 2

Patient / Family Medical History Table 3

Charlson Comorbidity Table 4

Breast related outcomes Table 7

Colorectal related outcomes Table 9

For persons diagnosed with cancer, we will also obtain data from the treating hospital tumor registry if possible.

*Additional sources of medical information*: For subjects who received treatment at the Lifetime Cancer Screening Center or the H. Lee Moffitt Cancer Center, we will supplement primary care clinic chart reviews with additional sources of information. Clinical records from Moffitt (PowerChart) will be reviewed for data elements that are missing from primary care records. In addition, for subjects who received cancer care at the Moffitt Cancer Center, the Moffitt Cancer Registry will be queried to determine all aspects of subjects’ first course of cancer treatment.

The following instruments were later added to be conducted among study participants. These are self-reported surveys to evaluate psychosocial determinants of health. These surveys are to be administered shortly after diagnostic resolution and, if the patient is diagnosed with cancer, three months following the initiation of treatment:

1. PHQ Depression
2. Perceived Stress Scale
3. Cancer Worry Scale
4. Cancer Risk Scale
5. Cancer Response Efficacy Scale
6. Brief COPE
7. Pearlin Mastery (Perceptions of Control)
8. ISEL Social Support
9. Use of Tobacco/Alcohol
10. IPAQ Physical Activity
11. Preventive Health Behaviors
12. FACT B or C
13. Multidimensional Fatigue Symptom Inventory

**Process measures of navigation**

The following information will be collected to better understand the processes of navigation.

1. *Navigator demographic survey* – This instrument will be administered to all navigators to describe demographic characteristics of navigators. See NOVA Data Dictionary Table 5 for description. Please note that this information will be collected by NOVA for program evaluation and will not be available or used by project staff at the Moffitt Cancer Center.

2. *Navigator tracking log* – A log will be maintained by each navigator documenting the processes of patient navigation. The information that will be collected is documented in the NOVA Data Dictionary (Table 11). This log will document the type, length, and number of encounters navigators have with patients (Table 11a), the barriers to care that patients self-identify (Table 11b), and the actions navigators take to address patient barriers (Table 11c). In addition, data will be collected in such a way that specific navigator actions and work times can be correlated with barriers that were addressed (Table 11d).

3. *Patient navigator performance checklist* – The performance checklist will be administered by the navigator’s supervisor at least two times each year. The information to be collected in this checklist is described in Table 13 of the NOVA Data Dictionary.

4. *Clinic/Navigator Satisfaction Instruments*. - To assess constructs that relate to satisfaction with the navigator system, we will administer satisfaction instruments to clinic staff, primary care providers, and patient navigators at least yearly.

| **Navigator system related data** | **Data Source** |
| --- | --- |
| Demographics of Navigators | Navigator survey |
| Age | Navigator survey |
| Gender | Navigator survey |
| Race-ethnicity | Navigator survey |
| Primary language | Navigator survey |
| Secondary languages | Navigator survey |
| Socioeconomic status | Navigator survey |
| Educational degrees (field, degree) | Navigator survey |
| Relevant training / experience | Navigator survey |

| **Clinic / Navigator Survey** | **Data Source** |
| --- | --- |
| **Clinic staff** |  |
| Acceptance of navigator system by staff | Clinic / navigator survey |
| Acceptance of navigator system by PCP’s | Clinic / navigator survey |
| Perceived strengths of navigator system | Clinic / navigator survey |
| Perceived weaknesses of navigator system | Clinic / navigator survey |
|  |  |
| **Navigator staff** |  |
| Description of training | Clinic / navigator survey |
| Description of key resources used | Clinic / navigator survey |
| Patient load | Clinic / navigator survey |
| Time Diaries for Imputing Navigator Costs | Clinic/navigator survey |
| Perceived maximum patient load | Clinic / navigator survey |
| Job satisfaction | Clinic / navigator survey |
| Perceived strengths of navigator system | Clinic / navigator survey |
| Perceived limitations of navigator system | Clinic / navigator survey |

**D.4.6a Contamination**

Contamination is a concern to the extent that patient navigation services are provided to control sites. Contamination will reduce the apparent efficacy of patient navigation, biasing this estimate toward the null. We will take the following steps to reduce contamination. First, we will conduct site assessments and interviews with key staff to understand the current systems of patient navigation at control clinics. We will educate clinical providers and office staff at control sites about the nature of the study and the need to use existing systems of care rather than attempting to use navigator services at intervention clinics. We will remind control sites that they will receive navigation services later in the study. We will also offer control clinics basic educational materials (brochures etc.) outlining resources that are available in the community. If control patients, providers, or office staff seek out navigation services at intervention clinics they will be referred to appropriate clinical staff at their existing clinic. We will also monitor the extent of contamination of navigation services at control sites. Our community advisory board and focus groups will also make recommendations to reduce contamination.

**D.4.6b Referral Bias**

A comparison of outcomes between navigated and control subjects may be biased if providers selectively refer subjects into the study. Providers may selectively refer patients that they judge to be in most need of navigation (i.e. those subjects having the most barriers to care). This would tend to bias results toward the null. To assess whether selective referral occurs and the magnitude of its effect, we will undertake the following assessment.

First we will conduct a brief survey of providers that inquires whether providers are more likely to refer selected patients for navigation and if so what are the patient characteristics associated with greater likelihood of referral. This self administered survey will be distributed to all providers (n~35) and responses summarized. Descriptive statistics will be used to describe provider referral patterns but no hypotheses will be formally tested.

The second assessment of potential referral bias will be conducted using medical record abstraction. We will identify patients meeting eligibility criteria for navigation using administrative data sources (billing records, referral logs, tickler systems). Medical records will be abstracted to assess study outcomes and allow comparisons between eligible subjects that were referred for navigation and subjects who were eligible but not referred. We will compare these two groups in regard to the main study outcome (time to definitive diagnosis) and by patient characteristics. We hypothesize that providers will selectively refer subjects having more barriers to care). We will abstract the medical records of up to 100 subjects per intervention clinic (700 subjects total). The sample size specified will provide >80% power to detect a standardized effect size of 0.15 in comparing outcomes between referred and non-referred intervention subjects.

**D.4.7.a** **Outcomes**

The primary outcome for this study is the time interval (in days) between abnormal finding suspicious for cancer and resolution. The onset of this time interval is the date of the abnormality meeting eligibility criteria *(Breast:* date that abnormal mammogram/US/MRI read by radiologist, date of abnormal clinical examination finding. *Colorectal:* date of abnormal screening [date sigmoidoscopy/colonoscopy performed, date FOBT interpreted], date that abnormal imaging test read by radiologist). If more than one abnormality meets eligibility criteria the onset of this time interval will begin with the earliest abnormality. Resolution is defined as the date that a definitive diagnosis is rendered regarding the initial abnormality. For breast, resolution may be indicated by definitive pathologic/cytologic findings (for women who undergo biopsy), definitive radiologic diagnosis, or by expert opinion (from breast surgeon). For colon, resolution will be defined by the date that pathologic interpretation of a biopsy specimen is rendered (if biopsy is performed) or by the date of diagnostic colonoscopy / radiologic imaging if no biopsy is performed. If more than one abnormality meets eligibility criteria resolution will be defined for the most clinically serious abnormality diagnosed (e.g. invasive cancer>>cancers in situ>>benign pathologic findings>>definitive radiological tests)

**D.4.7.b Statistical Analysis and Study Power**

The statistical analyses will focus on those subjects receiving an abnormal or suspicious mammogram (BIRAD 0, 4, or 5) and those with abnormal FOBTs or abnormalities detected on sigmoidoscopy (see D.2.1). Those in the Navigator intervention groups will be compared to those in the control groups with respect to two outcomes. For patients with breast or colorectal abnormalities, the primary outcome will be time from detection of abnormality to definitive diagnosis, measured as a continuous variable. In addition, we will assess the proportion of patients who successfully complete an appropriate diagnostic evaluation. The records of each subject with such an indication for follow-up will be examined as described in D.3.3.

Study power is based on the following factors. The primary endpoint is ‘Length of time’ (LOT=time from screening to definitive diagnosis). Suppose LOT follows a normal distribution (mean=30 days, “std” = standard deviation). Then, to detect the difference in LOT between two groups (navigator versus control groups), a two-sample t-test will be used. The required minimum sample size to achieve 80% statistical power is calculated based on the t-test and illustrated in Figure 1.

For example, group sample sizes of 29 and 29 (total 58) achieve 80% power to detect a difference of 10 days between the null hypothesis that both group means are 30.0 and the alternative hypothesis that the mean of Navigator group is 20 days with estimated group standard deviations of 15.0 each and with a significance level of 0.05 using a one-sided two-sample t-test. However, to detect a difference of 5 days with the same standard deviation 15.0 between the two groups, one would need at least 112 samples for each group (total 224).

Figure 1. Required sample sizes for each group based on the expected mean of navigator group by the standard deviation. For the control group, the mean value of LOT was set up as 30.0. The standard deviations for both groups are assumed to be equal. α=0.05 (one sided: mean 1 > mean 2).

Although we do not expect attrition of clinics, we have assessed power the possibility of between 6 and 8 participating clinics enrolling about 100 subjects each (a total of 600 - 800 subjects). Depending on the intracluster correlation coefficient (ICC) and the design effect, however, there may be effectively fewer subjects enrolled in this study from a statistical perspective. Assuming the intracluster correlation coefficient (ICC) to be in the range of 0.01 through 0.04, the effective sample sizes will be obtained as follows,

| # of total clinics | Actual Sample Sizes | ICC | Design Effect | Effected Sample Sizes |
| --- | --- | --- | --- | --- |
| 8 | 800 | 0.01 | 1.99 | 402 |
| 6 | 600 | 0.01 | 1.99 | 302 |
| 8 | 800 | 0.02 | 2.98 | 268 |
| 6 | 600 | 0.02 | 2.98 | 201 |
| 8 | 800 | 0.04 | 4.96 | 161 |
| 6 | 600 | 0.04 | 4.96 | 121 |

Therefore, given our expected sample sizes (600-800 subjects) even under the most conservative conditions (low design effects or ICC values, and relatively high population standard deviation), we will expect the desired statistical power will be achieved.

We will also conduct a number of secondary analyses in an exploratory fashion. For those with a suspicious screening result, we will compute the Kaplan-Meier curves for time from the suspicious result to definitive diagnosis (positive or negative) for each randomized arm. For those with positive diagnoses, we will compute Kaplan-Meier curves for time to onset of therapy for each randomized arm from both suspicious screening result and definitive diagnosis. In each case, we will estimate the standard error of these estimates with Greenwood’s formula and produce 95% confidence bands for these curves using the method of Hall and Wellner. The log rank test will be used to compare the two randomized groups. These comparisons will be at the two-sided 0.05 significance level.

Degree of adherence to NCCN standards will be assessed by an independent assessor blinded with respect to randomized arm and rated as 1 = complete adherence, 2 = substantial adherence, or 3 = poor adherence. The two randomized arms will be compared with respect to these adherence ratings using Wilcoxon’s Rank Sum Test.

All comparisons of the Navigator Program to the control group will be based on the ‘intent to treat principle’. That is, all patients who give consent and are registered on the study will be analyzed according to the randomized group assignment without regard to their degree of participation. Additional analyses, limited to those assigned to the Navigator Program, will explore the effects of degree of participation on the study endpoints. These analyses will incorporate participation ratings of 0 = no participation, 1 = some participation, 2 = substantial or complete participation that will be given to each patient by the Navigator assigned to that patient. Although outcome data on those patients refusing to consent in the study will not be available, we will compare those refusing to those agreeing to participate with respect to demographic variables such as age, race, gender, etc.

### All statistical analyses will be performed using SAS® software (version 9.1). Data will be computerized using an Excel database designed by the study biostatistician with input from the P.I. This database will be transmitted monthly to the biostatistician who will convert it to a SAS database using a commercial product designed for that purpose, STAT Transfer. The biostatistician will write and run programs designed to detect data errors or inconsistencies. Such problems will be discussed with the P.I. and corrected.

### **D.4.7.c Cost-effectiveness Analysis**

The cost-effectiveness analysis (CEA) requires a careful, prospective enumeration of the incremental costs of conducting the Navigator Program, net of the costs incurred in conducting the research protocol[86]. The cost enumeration will be predicated on the economic concept of opportunity cost and will take both payer and societal perspectives[87]. The opportunity cost criterion mandates that the economic value of all human and capital resources utilized in conducting the navigator intervention be measured in dollar terms, including the implicit time costs of uncompensated or volunteer workers who may complement the activities of paid navigators. Human resource costs will be gauged by quantifying the time inputs of all persons utilized in conducting or producing the intervention, and then valuing this time by observed market wage rates adjusted for fringe benefits and inflationary trends. In most cases, capital costs will be estimated by the investigator team on a “step down” basis from information on human resource inputs. Either direct observation and/or the completion of time diaries by navigator staff will be employed to collect time input data.

The perspective taken in constructing the cost variables will determine the range of inputs encompassed by each measure. From a payer perspective, only inputs that are potentially reimbursable under a standard range of third-party arrangements/plans will be included; from a societal perspective, all inputs, irrespective of whether they are reimbursable, will be included. For present purposes, the most important difference between the two perspectives is whether the time inputs of breast and colorectal cancer patients themselves are valued and then added to the societal cost numerator term. Since the Patient Navigation Program aims to reduce the time individuals spend in the pursuit of successful cancer treatment, the value of the time between abnormal screen and diagnosis or definitive treatment provides a convenient proxy measure of the economic input of patients. Sufficient information about the time paths of navigated patients will be collected, and conventional methods can be used to value those times, e.g., by using the prevailing minimum wage rate in the US. Information on elapsed time between key events for non-navigated controls can also be obtained from medical records, and these times can be valued in economic terms in the same way. This dimension of the Navigation Program is of such crucial importance, however, that more detailed data about the time patients spend “producing or investing in” their diagnosis and treatment should be gathered. While such detailed data will be obtained from navigated patients by means of the follow-up interviews described previously, obtaining similarly detailed data on non-navigated controls is more problematic. The feasibility of surveying a small sample of non-navigated patients being diagnosed and treated contemporaneously at the Moffitt Center to obtain such information will be assessed during year 1 of the proposed study. If feasible, comparison data on time inputs of both navigated and non-navigated patients can be used to estimate the costs of the intervention from the societal perspective.

The incremental cost/incremental effectiveness ratio of the intervention will be computed. From a payer perspective, the incremental cost numerator term of the navigator intervention is simply the expected (mean) cost per patient, because the cost in the absence of the intervention is taken, by definition, to be zero. From a societal perspective, however, there is a cost term even in the absence of the intervention, viz., the incremental cost numerator term is the algebraic difference in the estimated values of patient time inputs between case and comparison patient groups. A negative value, all other things equal, would imply that the Navigator Program produces cost “savings.”

Incremental cost figures, however, must be related to, or standardized by, differences in attributable outcomes in order to admit to policy-relevant interpretation. Several variants of the incremental cost/effectiveness ratio (ICER) will be computed for purposes of drawing cost-effectiveness conclusions[46]. First, statistically significant differences in short-term outcomes and process indicators that are yielded by the navigator trial can be used as denominator terms, and the computed ICERs based on these measures can then be compared to similar ones found in the published literature. To illustrate, the proportional increase in the number of recently diagnosed breast cancer patients completing adjuvant therapy that is attributable to the navigator intervention can be used as a denominator term; the corresponding ICER provides an estimate of the cost of increasing the probability of such guideline adherence by a given amount. This ICER, however, does not admit widely to comparison and thus may prove difficult to interpret. Yet, there are a variety of ICER values in the literature for closely allied breast cancer interventions such as screening outreach. Conceptually, the cost-effectiveness ratios of interventions designed to increase detection and treatment at different points in the natural history of the disease should be roughly equal. We propose to conduct a comprehensive review of the existing literatures on breast and colorectal cancer diagnosis and treatment in order to compare ICERs across the set of feasible alternatives and, thereby, judge the economic worth of the navigator intervention.

The second approach or variant for appraising ICERs overcomes the limits of the first by translating outcome indicators into more conventional effectiveness measures that admit to a much wider set of comparisons. Primary focus in this regard will be to translate differences in clinical outcomes and short-term survival data, when they become available, into life-year (LY) and quality-adjusted life-year (QALY) equivalents. This approach is now commonplace in the health services research literature, and is illustrated concretely in recent papers by one of the investigators[46, 88]. Translating significant differences in outcome indicators into differences in LYs or QALYs permits comparisons of the economic value of the navigator intervention to a host of other health-related interventions, both in and beyond the cancer field. By a line of now familiar reasoning, health-related interventions that produce the lowest (incremental) cost per life-year or quality-adjusted life-year ratios should be accorded higher priority for funding and implementation. The “lowest” ratio in this context is often further subdivided between dominant interventions-- those with both lower incremental cost numerators *and* higher incremental LY or QALY-gain denominators--and semi-dominant interventions--those with either a lower numerator *or* a higher denominator, but not both.

Because the Navigator Program is designed to *complement* the full range of efforts to diagnose and treat cancer, we propose also to carry out a third approach to evaluating its cost-effectiveness. In brief, complementary interventions should be evaluated by comparing two ICERS, one referring to treated disease in the absence of the intervention, and the other treated disease in the presence of the intervention. A cost-effective complement should lower the treatment ICER and, in effect, “pay for itself;” see[44, 89] for details and illustrative computations. This approach has the added advantage of introducing treatment costs more explicitly into the analysis. Operationally, cumulative costs of treatment by stage of disease and type of treatment regimen can be computed by distilling the results of national studies, valuing the treatment patterns gleaned from medical chart abstracts and/or exploiting administrative claims records of some study subjects, viz., those treated at the Moffitt Center. (See[90] for an illustrative analysis drawing on local claims data and a discussion of problems associated with censored cost data). Outcome differentials associated with stage and treatment regimen can then be used to compute LYs or QALYs and, correspondingly, the ICER. Thereafter, a second ICER will be computed that adds (algebraically) the costs of the navigator intervention to the cost numerator term and the LY or QALY gain to the denominator term. If the complementary intervention is cost-effective, this second ICER will be lower than, or equal to, the first. If it is higher than the first, the conclusion is drawn that the navigator intervention is not cost-effective.

The program’s steering committee later decided that, in lieu of the process described above, the cost effectiveness analysis was to be coordinated by NOVA under the direction of Scott Ramsey, and that each of the sites involved in this study was to assist with tasks such as finalizing, pilot testing, and completing survey instruments.

**D.5 Phase III: To disseminate the findings from this research outside the Tampa Bay region.**

**D.5.1 General Strategy**

Translating research findings into evidence-based services is a critical step in addressing health disparities. This proposed project will determine the effectiveness of a Patient Navigator Program under experimental conditions. In the last year of the project, we will determine whether it is feasible to implement navigator systems beyond the experimental setting. Our analysis will therefore assess the extent to which navigating systems can be implemented more broadly, without direct support of research staff, and the degree to which they are able to emulate key aspects of the original system. Although dissemination plans cannot be finalized in detail at this time, and will not be finalized without input from the Community Advisory Committee, several general issues can be presented here that will guide Phase III. The most important issue is that the dissemination is informed by the study outcome findings. These include results on the effectiveness of the Patient Navigator Program on timeliness of care, quality of care, cost-effectiveness, and patient satisfaction. We anticipate that the Patient Navigator will have a significant positive effect on each of these study endpoints. Our dissemination plan would include the development of an evidence-based protocol on training of Navigators, recruitment of volunteers to perform this important service, and a systems-based approach to implementation in a variety of clinical care environments.

In addition to the study outcome data and cost-effectiveness analysis, we will also obtain data from two key stakeholders for long-term dissemination: participants and providers. Qualitative methods applied in the community research setting can provide valuable insights for designing effective program approaches. A variety of qualitative data will be collected to inform the dissemination plan. These include focus groups with medical staff (nurses, doctors, desk attendants, etc) at the community clinics, the Navigators, and the research coordinators. Questions will include feedback provided by the patients (to supplement the patient satisfaction structured follow-up), changes in the clinic following the intervention, and suggestions to improve the process. The Community Advisory Committee will be used as a source of input, and will be dedicated to the dissemination plan in Year 05. A unique strength of the proposed study is the collaborative partnership with Health Choice Network (HCN), one of the strongest community health center networks in the nation. More on HCN is provided in the next section. Although it is beyond the scope of the current study to conduct a full trial of the dissemination, it is anticipated that the focus group work will include member centers of HCN outside the Tampa Bay region, and that some pilot work may be conducted.

### **D.5.2 Health Choice Network**

As reviewed in C.5, Health Choice Network (HCN) is a community-based, not-for-profit, 501(c)(3) organization formed by community health centers (Centers) in Miami Dade in 1994 to improve quality and efficiencies of care through operating integrated systems and programs serving all members. HCN’s member Centers provide high-quality, comprehensive primary and preventive care to low-income, underserved populations**.** Together, HCN members provided care for more than 180,000 patients in Florida in 2003. As a pioneer in networking of Centers, HCN is highly visible in the community health field and will be an effective advocate for dissemination successful patient navigation models. Thus, HCN is well-positioned to be the primary driver of Phase III.

**D.6 Timeline**

**
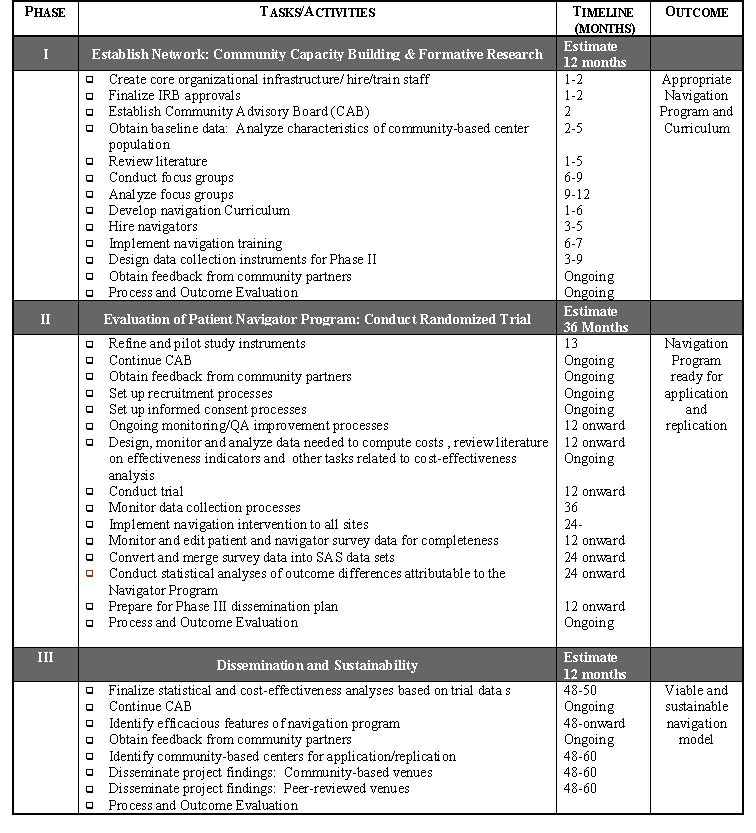
**

**E. Human Subjects Research.**

**E.1. Protection of Human Subjects.**

*E. 1.1.Human Subjects Involvement and Characteristics.--*

We are proposing a five-year project to develop and evaluate an appropriate and effective Navigation Program according to timeliness to diagnosis, appropriateness of care, satisfaction and quality of life. The study will be conducted at community-based centers/clinics that focus on serving medically populations. These sites include the Suncoast and Tampa Bay Community Health Centers (both federally qualified clinics) and Tampa General Hospital ambulatory clinics (public hospital). Subjects will be enrolled at both control and intervention sites. The research project will not affect the standard medical care that patients will receive. The intervention will only change the manner in which follow-up is provided, that is, adding a focused cancer navigator to the array of resources and outreach services that currently exist at these community-based centers. Control site practices will continue their usual efforts to promote tracking and follow-up of abnormalities and as part of this project, all sites will receive the Patient Navigation Program once it has been initially evaluated.

There are three phases to this study. In Phase I, a maximum of 70 subjects will be enrolled in focus groups or in-depth interviews and an anticipated 700 charts will undergo abstraction for baseline de-identified data. In Phase II, at most 1600 will be enrolled. In Phase I, information from patients’ medical records will be used to determine baseline data. For this reason, IRB and HIPAA issues are relevant for patients whose medical records are randomly chosen for review. We will request from the IRB a waiver of informed consent for medical record review and similarly a waiver of HIPAA authorization for medical record review in Phase I. We believe the project meets the four conditions necessary for waiving informed consent (45 CFR 46.116 (d) for this aspect of the study (chart review).

All members of the project team have experience in conducting community-based education and research and are sensitive to community needs and recognize historical and ethical issues relating to vulnerable populations. The project team will work closely with our community members to plan activities to ensure that they are carried out in the highest respectful and ethical manner. The entire proposal and proposed activities will be subjected to internal and external review by Moffitt’s Protocol Review and Monitoring Committee (PRMC), and the University of South Florida’s Institutional Review Board (IRB) as required and appropriate, (e.g., focus groups outlined in needs assessment, interviews, surveys). All members of the investigative team have completed the NIH computer-based training course on Protection of Human Research Subjects and the relevant continuing education course per federal regulations and are in compliance with institutional and University of South Florida Institutional Review Board (IRB) requirements. For all proposed collaborative activities collection of baseline data and focus groups in Phase I, no personally identifiable information will be linked. In Phase II, however, we will collect personal data to follow up with participants for later assessment of intervention impact on outcomes, for example. All such data will be kept in locked storage and only study staff and investigators will know identities of participants.

*E.1.2. Sources of Materials.--*(To be used for research purposes only). Subjects will provide information about themselves in focus groups and interviews and offer their opinions in Phase I. Focus groups (Phase I) and in-depth interviews will be audio-recorded, transcribed verbatim, manually coded and then reviewed by each member of the research team. Some of the interviews in Phase II-III may also be audiotaped and field notes kept from subject responses. The documents (consent forms, field notes, subject responses, audiotapes and transcripts) will be kept confidential. Once a subject agrees to be in the study (Phase II), we will assign ID numbers unlinked to his/her identity. Data will be recorded under that ID number. All documents, transcripts and tapes will be stored in a locked file cabinet in the Cancer Control Research Department at the Moffitt Cancer Center. All of this data is for research purposes only, and will be kept in strict confidence and protected with the utmost care.

*E.1.3. Potential Risks.--* This project poses minimal risk to participants.

1. The information that will be collected from chart abstractions is not of a sensitive nature, and would not reasonably put patients at risk. All information will be kept confidential and will be used for research purposes only. To further protect privacy, information from medical record reviews will be identified by a research ID number that will be linked to patient’s medical record number on a separate file kept on a password protected computer within a locked and supervised office at Moffitt. All research personnel involved in this study will have undergone appropriate training in IRB and HIPPA issues, as mandated by the University of South Florida.

2.There are no known physical risks posed by the proposed activities. We are using standard methods and interview techniques in the collection of data and standard procedures for follow-up of clinical abnormalities. Study participants will not encounter risk greater than those ordinarily encountered in daily life and will only be asked to provide answers to open ended and semi-structured questions related to their perceptions of care and satisfaction. However, if new risks surface, they would be detailed accordingly in the required IRB forms and subsequently communicated to participants in an understandable fashion in their preferred language.

**E.2. Adequacy of Protection Against Risks**

*E.2.1. Recruitment and Informed Consent.--* The collaborative investigative research team brings considerable expertise working in community settings and is experienced in focus group and interview methodology. Participants will be recruited at the level of the community-based site.

Potentially eligible subjects for Phase II will be identified from intervention and control clinics using methods described in **D.4.3**. Our process of obtaining informed consent will then be as follows. Using the mailing address obtained from clinic records, we will send individuals who are eligible an introductory letter that briefly describes the study and includes a toll-free number for individuals to call within one week if they do no wish to be contacted. The mailing will also contain two copies of the study informed consent document, two copies of the HIPAA authorization, and a stamped return envelope. At least 2 weeks following the mailing of the introductory letter, potential participants will be contacted by telephone and provided a brief description of the study in their preferred language (Spanish or English). We have bilingual/bicultural research members as part of our collaborative team. Key elements in this discussion are to inform subjects of the purpose of the present study, possible risks and provisions for them, lack of guarantee of benefit, confidentiality of responses, voluntary nature of the study and the right to withdraw or refuse any questions. It will also state that if they decide not to take part that it will not affect their relationship to the health clinic. An individual who states that he/she does not know how to read well will be assisted by bilingual and bicultural members of the research team on an individual basis: the form will be read aloud to them.

Verbal consent will be obtained from all subjects for their participation in Phase II and recruited participants will be asked to mail back 1 signed copy of the informed consent and HIPAA authorization in the pre-stamped, addressed envelope. If the informed consent document has not been received within 2 weeks of the date of the phone call, the participant will be contacted by telephone up to two more times to be reminded to mail the completed informed consent forms. All informed consent statements will be approved by Moffitt’s PRMC and USF’s IRB. Key elements in this discussion are to inform subjects of the purpose of the present study, possible risks and provisions for them, lack of guarantee of benefit, confidentiality of responses, voluntary nature of the study and the right to withdraw or refuse any questions.

Subjects will be informed that if they agree, they will be:

**Phase I:** (N= maximum 70)

Asked to take part in a small group discussion for about 1 l/2 hours or participate in an in-depth interview. During this time, they will be asked questions about their experiences in getting health care in their community (patients) and or their perceptions about assessing health care in the community (community informants).

Phase II: (N=1600)

Agreeing to have their medical records abstracted to examine the care they received and its timeliness. For a randomly chosen subgroup, they will be asked to take part in brief interviews by telephone of about 20 minutes. During this time, each subject will be asked a series of questions about their satisfaction with the health care they received, and their perceptions of their health-related quality of life.

Clinic providers

We will also obtain informed consent from the medical providers (physicians, ARNP’s, PA’s) at all participating clinics. The nature of the study will be explained and the study requirements for themselves and their patients will be outlined.

*E.2.2. Protection Against Risks.--*Potential risks are outlined below as well as ways to minimize such risks.

Risks: A concern could be raised that a loss of privacy could occur during the focus group (Phase I) if personal information is shared with others. In Phase II, the unit of randomization is at the clinic level and thus subjects in the control group will not be receiving the patient navigator intervention. However, patients at all clinics do receive follow-up and tracking of abnormalities through existing protocols according to their established standards of care. However, in consideration that this intervention could be efficacious, we do plan to offer the Patient Navigation Program to all sites after the initial evaluation has been conducted in the first two years. Thus, all patients at all sites will be exposed to the Patient Navigation Program at some point during the project.

Minimization: Within focus groups, all participants will be told at the start of the focus groups that information shared within the group should remain within the group. Subject data will be available only to research staff involved in this project. Data will be kept in locked filing cabinets in locked research rooms. Identifying information will not be reported. With regard to patients who are randomized to the control group, they will continue to receive standard care and outreach and follow-up, which is part of the standard protocols at the community-based centers. Once evaluated under controlled conditions, the Patient Navigation Program will be offered at all sites.

Risk: Exchange of information from one institution to another (Moffitt Cancer Center and the community-based centers).

Minimization: All data exchanged electronically from one institution to another and among team members will be in aggregate form with no identifiers.

Risk Chart abstractions conducted at six months or 12 months follow-up may identify patients for whom recommended care has not yet been received.

Minimization: Research assistants will compile listings of all patients for whom chart abstractions suggest that recommended care has not been received. These listings will be forwarded to the patient’s primary physician for review. Information that will be forwarded will include patient’s name, medical record number, and a listing of the targeted health services that were recommended but not received according to information obtained in the medical record abstraction.

**E.3. Potential Benefits of the Proposed Research to the Subjects and Others**

The proposed five-year project involves collaboration with community-based health centers in Hillsborough County. It is expected that the people who are served by these organizations may take part in and benefit from health we jointly conceive, plan and implement and that a greater understanding about ways to reduce cancer health disparities might result from project activities. There is no guarantee of benefit to participants based on study participation. However, information gained from the study should be helpful in knowing how to best communicate important information needed to get services and care that may be helpful to the future health of other community members. The risks involved in the study are minimal, but the benefits to the individual subjects and to society in general are considerable.

**E.4. Importance of the Knowledge to be Gained**

We anticipate that participants in our study will benefit by gaining new knowledge that will empower them for assessing required health services action and allow them access to more timelier care. Potential risks are minimal through adherence to strict protocols for protecting identity, and potential timely tracking of abnormalities through tracking and follow-up by navigators. Collaborations with community partners to create relevant programs and services will serve to benefit the wider community through the definition of an effective health care delivery interventions aimed at reducing the unequal burden of cancer in our local community. All research team members (applicant organization and collaborating organization) will receive training in research ethics. The research team has considerable experience managing risk with this type of research project. Confidentiality will be maintained by using subject numbers on data, rather than subjects' names.

### **E.5. Women and Minority Inclusion in Clinical Research**

All subjects in this study will be recruited from community-based clinics known to serve medically underserved populations. We are committed to the inclusion of subjects regardless of gender, race or ethnicity. No individual is excluded from the programs of the center or its research protocols on the basis of gender or racial/ethnic status.

*E.5.1 Inclusion of Women.*-- Our recruitment strategy is designed to produce a sample of patients representative of those receiving care at the community-based clinics identified in this application in Hillsborough County. Given the population-based nature of primary care, this should produce a sample of participants that are at least representative of Hillsborough County as a whole. Because women tend to be overrepresented in physician office derived samples and because abnormalities suspicious for breast cancer occur almost exclusively among women (unlike colorectal abnormalities), we anticipate that the proportion of women in our sample will be greater than 50%.

*E.5.2 Inclusion of Minorities.*-- Given our recruitment strategy and sampling scheme within practices, we anticipate that minority participation in the project will be at least representative of the overall population in Hillsborough County and closer to that observed at the community-based clinics. Based on 2000 US Census estimates for Hillsborough County for adults and estimates of the racial/ethnic composition at our community-based clinics, we anticipate that our sample will consist of the following racial / ethnic distribution: Hispanic 29%; Black 22%; White 45% and other 4% (Native American, Asian).

*E.5.3 Inclusion of Children.*-- It is expected that the intended population primarily will be adults, which represents the group most at risk for cancers, e.g., breast, colorectal, and most amenable to cancer prevention and control efforts. However, we might expect that women younger than 21, that is, *children* according to NIH guidelines, might take part in some screening activities based on symptoms. Thus, we will include those 18 years of age and older as appropriate and will employ appropriate informed consent procedures.

**E.6. Data and Safety Monitoring Plan**

Due to the minimal risk to participants, we will not create a formal data safety monitoring board. We will take the following steps, however, to insure patient safety. First, all members of the research staff will undergo required IRB training and all participating clinical sites will complete Single Project Assurances for the ethical conduct of research. Any adverse event will be promptly reported to the USF IRB. The principal investigator will submit a continuing review summary to the USF IRB annually. During all phases of the project and during implementation of the intervention, project staff will make contact with participating clinical sites at least weekly to inquire about concerns or problems relating to patient safety. After the intervention has been fully implemented research staff will make such inquiries monthly. The principal investigator and staff will review with research staff/navigators on a monthly basis any issues relating to patient safety. Finally, the project will has in place a community advisory board to provide advice to the investigative team regarding practicality and acceptability of intervention methods and materials, and will also consider issues regarding patient safety. The advisory board will meet twice yearly during each year of the project.

##### Issues of data integrity and confidentiality will be supervised by the principal investigator and statistician jointly. These persons will insure that data confidentiality is preserved and that patient identifiers are destroyed at the earliest opportunity. There are no plans for an interim analysis or early stopping rules. We will also register the intervention project in a suitable clinical trials registry at the start of the project. Each protocol receives Scientific Review and Institutional Review Board (IRB) approval prior to activation. This protocol and informed consents, will meet the applicable guidelines of these scientific review bodies. Written evidence of IRB approval must be submitted to the Protocol Monitoring Committee of the Cancer center before accrual to any study is allowed. The next date of approval is calculated by the database, ensuring that investigators do not continue enrollment if annual re-approval is not provided. This database also enables the Protocol Monitoring Committee to immediately halt accrual, as needed, by suspending registration for a particular study. Once informed consent has been obtained, this information is documented during the registration process and on initial study forms submitted to the Operations Center. Specifically, in this study, our database will be password protected and only our research team members will have access to it. Any transmission of the data will have identifiers expunged. The computer that will house the database is part of a network that is backed up daily by the Cancer Prevention and Control Division's Research Computing Support staff. The network also makes use of the latest in firewall technology to prevent unauthorized access or tampering and keeps up-to-date virus protection on each computer as well as the network.

**E.7. Data Sharing Plan**

The Patient Navigator Research Project is a cooperative multisite study funded by the National Cancer Institute. Individual research sites will work in partnership with NCI to assess outcomes of navigation both locally, and nationally. Therefore a requirement of this cooperative funding arrangement is that all participating sites will submit data to the NCI to allow a national evaluation of the program. Data elements to be submitted will be defined by the national steering committee. The data submitted will not contain elements that would uniquely identify individual subjects. The submitted data will be used by NCI for research purposes only and will not be submitted to other parties without approval.

1. **VERTEBRATE ANIMALS**

N/A

**LITERATURE CITED**
